# Projection or admittance? Presupposition accommodation and the Karttunen calculus

**DOI:** 10.1007/s10988-025-09431-1

**Published:** 2025-06-14

**Authors:** Yoad Winter

**Affiliations:** Utrecht University, Utrecht, The Netherlands

**Keywords:** Presupposition, Admittance, Inference, Formal semantics, Proviso problem, Accommodation, Projection

## Abstract

This paper examines two approaches to presuppositions: one viewing them as inferences projecting from sentences under negation and other logical operators, and another defining them as admittance conditions of utterances. Neither approach fully accounts for the “proviso problem”, which arises when a sentence’s presuppositional inferences are logically stronger than its necessary admittance conditions. To address this challenge, we propose a calculus of a trivalent logic that formally distinguishes between admittance and projection, extending Karttunen’s dynamic, logical form-based analysis. The resulting framework enables a simple pragmatic strategy: presuppositional conclusions are accommodated unless overridden by a contextually likelier admittance condition. We provide evidence that this approach is empirically superior to methods that address the proviso problem using pragmatic strengthening.

## Introduction

Classical works on presuppositions view them as inferences that escape the scope of sentential operators, such as negation, conditionals and questions. By contrast, admittance-based approaches treat presuppositions as conditions that a context must meet for a sentence to be uttered felicitously. This paper argues for a unified semantic system that integrates both perspectives, and proposes a pragmatic principle for presupposition accommodation based on this system. We show that this principle provides a more adequate solution to the “proviso problem” than previous approaches that rely on pragmatic strengthening.

This work comprises two parts, which can be read independently. The first part develops the formal semantic aspects of the proposed system, emphasizing its distinctions from related frameworks. The second part applies these conclusions to the pragmatics of accommodation but does not require technical familiarity with the formal details. The remainder of this introduction outlines the background for both components of the paper.

The view of presuppositions as inferences was prominent in early truth-conditional analyses (van Fraassen, 1968) and it continues to guide much further work on the topic (Beaver et al., 2024). The admittance-based analysis was proposed by Stalnaker (1973) and Karttunen (1974), and received standard formulations in dynamic frameworks using possible world semantics (Heim, 1992; Nouwen et al., 2016). A priori, there is no contradiction between these two approaches. Furthermore, when analyzing semantic and pragmatic properties of language utterances it is empirically necessary to use both of them. To illustrate that, let us first consider the following example:
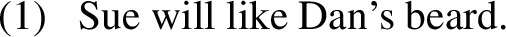


Putting matters of tense aside, from (1) we readily conclude the following sentence:
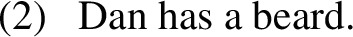


Like other entailments, we can describe this inference by observing that whenever sentence (1) is judged as true, so is (2). Unlike classical logical inferences, (2) is also judged as true when (1) is *false*. Equivalently, (2) is inferred, or “projected”, from the negation of (1), as well as from other complex sentences containing (1) (Chierchia & McConnell-Ginet, 1990). To avoid the theoretically-laden term “presupposition”, we call (2) a *presuppositional conclusion* of (1). In addition, statement (2) also has a pragmatic role in admitting utterances of (1). For (1) to be an acceptable utterance, statement (2) must be part of the common ground of the interlocutors, or else it must be silently accommodated by the hearer (von Fintel, 2008). Thus, we say that the presuppositional conclusion (2) is also a necessary *admittance condition* of sentence (1): it must be part of any discourse context where (1) is used felicitously.

Presuppositional conclusions and necessary admittance conditions do not always coincide in this way. For example, let us consider the following sentence:
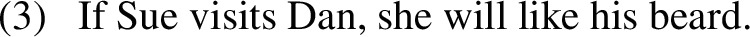


Out of the blue, speakers infer from (3) that Dan has a beard, similarly to (1). This qualifies (2) as a presuppositional conclusion of (3). However, (2) is not a necessary admittance condition of (3). To see that, let us suppose that (3) is uttered in the following context:
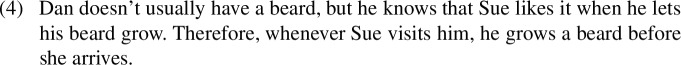


In the context of (4), hearers of sentence (3) accept it as felicitous. The statement (2) does not logically follow from the context in (4), nor can it be inferred from (3) when uttered in this context. Thus, the conclusion (2) that qualifies as “presuppositional” according to standard projection tests is not necessary for (3)’s admissibility. A more appropriate candidate for being a necessary admittance condition of sentence (3) is the following conditional:
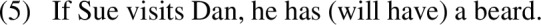


Any context like (4) that makes (5) true is expected to admit (3).

In most current semantic theories, sentences like (3) are treated by taking (5) to be (3)’s unitary “presupposition”. Notably, the dynamic semantics of Stalnaker and Heim treats contexts and presuppositions as sets of possible worlds, which correctly accounts for admittance phenomena. However, as pointed out by Geurts (1996), in cases like (3) the Heim-Stalnaker analysis does not directly account for presuppositional conclusions like (2), a problem that Geurts referred to as the “proviso problem”. A similar problem appears with trivalent theories of presupposition that rely on principles of the Strong Kleene truth tables (Kleene, 1952; Peters, 1979). Indeed, the problem that Geurts dubbed the “proviso problem” had been first discovered by Karttunen (1973, p. 188) as a problem for trivalent accounts.

To address the proviso problem of trivalent and possible world semantics, a common strategy is to strengthen the minimal admittance condition into a presuppositional inference. In semantics and pragmatics, there is a host of proposals as to how this strengthening takes place, with little consensus on its motivations and precise details. For discussion, see van Rooij (2007), Singh (2007), Schlenker (2011), Lassiter (2012), Fox (2008), Fox (2013), Mayr and Romoli (2016), Mandelkern (2016b), and Mandelkern (2018), among others.

But should our semantic theory aim at a unitary notion of presupposition in the first place? This paper argues for a negative answer on this question. As we will show, Karttunen’s (1974) analysis allows the core semantic mechanism to formally distinguish the admittance conditions of a sentence from its presuppositional conclusions. In Karttunen’s representation of logical forms, an admittance condition is satisfied if it is *logically entailed* by its local context. To see how this technical detail leads to different expectations than those of other dynamic analyses, let us reconsider sentence (3), representing its meaning using the following formula *S*:
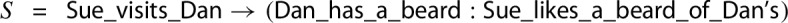


The notation ${(\textsf {Dan\_has\_a\_beard}\!:\!\textsf {Sue\_likes\_a\_beard\_of\_Dan's})}$ indicates that the statement ‘Dan has a beard’ is an admittance condition of (3)’s consequent (=*Sue likes Dan’s beard*). When this condition is satisfied by the consequent’s local context, the consequent asserts that Dan has a beard Sue likes. To obtain this local context, we need to update the global context of sentence (3) by conjoining it with *S*’s antecedent Sue_visits_Dan. Let us first consider a null global context, i.e., the tautological proposition $\top$, which represents a scenario where no prior constraints are imposed. In this context, the local context of *S*’s consequent is $\top \wedge {\textsf {Sue\_visits\_Dan}}$, i.e., ‘Sue visits Dan’, which does not entail that Dan has a beard. Accordingly, the admittance condition of *S*’s consequent remains unsatisfied. Karttunen (1974) did not expand on this point, but in the present analysis it gives us a straightforward account of why *Dan has a beard* is understood as (3)’s presuppositional conclusion when the sentence is uttered out of the blue.

Karttunen’s analysis focuses on cases where admittance conditions are satisfied. For our example, let us consider (3) in the global context (5), which is represented using the following formula *C*:
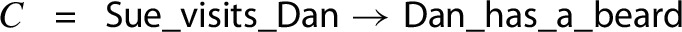


Updating *C* using *S*’s antecedent makes $C \wedge$ $\textsf {Sue\_visits\_Dan}$ the local context of *S*. By modus ponens, this local context entails, hence satisfies, the admittance condition ‘Dan has a beard’ of *S*’s consequent. Thus, in the context of *C*, sentence *S* has all its admittance conditions locally satisfied; hence, it has no presuppositional conclusions.

The analysis sketched above illustrates that the distinction between an admittance condition of a sentence and its presuppositional conclusion can be obtained by adding projection to Karttunen’s mechanism. To develop this idea further, the present paper introduces a logical system that we refer to as the *Karttunen calculus* (K-calculus). This system is based on the uniform “incremental” principles of the Strong Kleene trivalent truth tables, thus generalizing Karttunen’s proposal and avoiding some of its seemingly *ad hoc* properties. We argue that the K-calculus provides a sounder semantic basis than previous approaches that rely on a unitary definition of presupposition. To show that, we introduce a pragmatic strategy that we call *K-accommodation*. According to this procedure, presuppositional conclusions are the first candidates that hearers of a sentence *S* try to accommodate. If the strongest presuppositional conclusion *p* is among the pragmatically likeliest propositions that admit *S*, it becomes the only candidate for accommodation. This situation is exemplified in sentence (3) above, whose presuppositional conclusion *p*=(2) is accommodated when it is uttered in a null context. However, if there are pragmatically likelier propositions that admit *S*, hearers will accommodate one of these propositions rather than *p*. This may lead to inferences that are sometimes referred to as “conditional presuppositions”. For instance, from sentence (6) below, most hearers infer (7a) rather than (7b): 
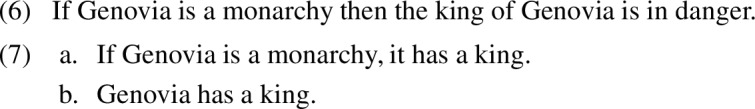


Upon hearing sentence (6) in a null context, deducing its presuppositional conclusion (7b) would violate the ignorance implicature about (6)’s antecedent. As a result, the logically weaker but pragmatically likelier admittance condition (7a) is accommodated.

The critical difference between K-accommodation and pragmatic strengthening of admittance conditions appears in cases where *a priori*, there is no pragmatic reason to prefer one of the candidate inferences. Under these circumstances, K-accommodation expects the presuppositional conclusion to be accommodated, whereas pragmatic strengthening expects the hearer to accommodate the admittance condition. Following Karttunen (1973, 1974), Geurts (1996) and Mandelkern (2016a,2016b), we show that in such cases, the presuppositional conclusion is indeed accommodated. We argue that this advantage of the K-calculus and the proposed K-accommodation strategy makes them empirically preferable to standard approaches augmented with pragmatic strengthening.

The paper is structured as follows: Sect. 2 introduces the K-calculus, highlighting its key differences from other trivalent approaches, specifically in the projection of presuppositional conclusions and their distinction from admittance conditions. Section 3 applies this distinction to K-accommodation, demonstrating its empirical advantages over pragmatic strengthening. Section 4 concludes. formally defines the incremental trivalent interpretation mechanism used in Sect. 2. employs this definition for proving the main logical results of this paper.

## Admittance and projection in trivalent systems: truth tables vs. $\text{ Karttunen }$ calculus

This section introduces the K-calculus, comparing its analysis of admittance and projection with standard theories of presupposition. Our starting point is trivalent truth-functional semantics (Kleene, 1952; Fitting, 1994). This framework provides a basis for analyzing presuppositions (van Fraassen, 1968) and extends naturally to dynamic approaches in possible world semantics (Stalnaker, 1973; Peters, 1979; Heim, 1992). In dynamic analyses, the formal presuppositions of an expression *exp* must be satisfied within its local context. This local context is derived by sequentially updating the global context of the sentence with the expressions that are compositionally processed before *exp*. Karttunen’s concept of satisfaction is similar to the Heim-Stalnaker analysis, but the two approaches differ in how they represent contexts and presuppositions. For Stalnaker and Heim, these are sets of possible worlds. Satisfaction between them is defined as set inclusion, with no explicit representation of failed presuppositions. By contrast, Karttunen defines satisfaction in terms of entailment between the logical forms of the context and the presupposition. Although Karttunen (1974) did not exploit this property, it allows us to track a failed presupposition and project it further if necessary.^1^

The proposed calculus uses this property to project unsatisfied presuppositions, enabling them to contribute to the derivation of the sentence’s presuppositional conclusions. This mechanism involves two generalizations of Karttunen’s proposal. First, Karttunen’s rules for updating local contexts lacked general motivation, which is provided here by the incrementality principles of trivalent semantics. Second, we present a unified framework for trivalent approaches, including Karttunen’s, as *projection calculi*—mechanisms that derive presuppositional conclusions from sentential formulas. This facilitates the comparison between the proposed K-calculus and other trivalent mechanisms.

With these theoretical preliminaries in place, we establish a general result that contrasts Karttunen-like calculi with trivalent truth tables. As we will show, the truth-functional account formally equates admittance with presuppositional inference, and this property carries over to dynamic approaches in possible world semantics. The K-calculus captures admittance similarly to truth-functional semantics. However, in cases revealing the “proviso problem”, the presuppositional conclusions that it projects are logically stronger than admittance conditions. This distinction between projection and admittance is crucial for our pragmatic proposal in Sect. 3.

The following subsections explore the different aspects of the proposal: Sect. 2.1 reviews the incrementality of trivalent semantics using Peters’s (1979) asymmetric version of Kleene’s truth tables, emphasizing its alignment of admittance with presuppositional inference. Section 2.2 defines a trivalent propositional language, setting the stage for calculi governing presupposition projection. Section 2.3 presents a projection calculus based on the Kleene–Peters (KP) tables, facilitating their comparison to the K-calculus, which is defined and demonstrated in Sect. 2.4. Section 2.5 establishes our main formal results, comparing the K-calculus with the KP tables. Finally, Sect. 2.6 explores a version of the K-calculus that treats symmetric projection from disjunctions.

### The Kleene–Peters (KP) connectives

This section reviews trivalent truth tables, showing how their analysis of presuppositions effectively identifies projection and admittance. Most trivalent approaches to presupposition rely on an “incremental” analysis: they disregard local presupposition failures once the interpretation of a full sentence has been determined by previously processed semantic values.^2^ To illustrate this idea, let us consider the following example: 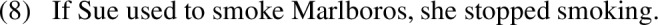


When we assume that Sue never smoked Marlboros, the incremental trivalent analysis of implication treats the conditional in (8) as true, just as a classical bivalent analysis.^3^ In this treatment, potential failures of the consequent’s presupposition in (8)—i.e., situations where Sue has never smoked—are ignored. This agrees with the “filtering” intuition about (8): the sentence doesn’t trigger non-trivial presuppositional conclusions. A similar treatment accounts for filtering with other connectives, as in the following examples: 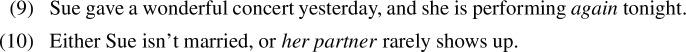


In (9), the trivalent analysis, like the classical bivalent analysis, treats the sentence as false if Sue didn’t give a concert, avoiding potential failures of the presupposition of *again*. Similarly, sentence (10) is treated as true if Sue isn’t married, preventing potential failure of the presupposition that Sue has a partner. In general: with all binary constructions, the incremental analysis ignores potential failures in the righthand operand if the value of the lefthand operand “determines” the result of the bivalent operation.

**Fig. 1:**
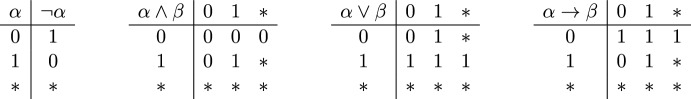
Kleene–Peters (KP) truth tables

Peters (1979) used a simple implementation of this incremental approach in his asymmetric version of the Kleene connectives. We refer to Peters’s connectives (Fig. 1) as the *Kleene–Peters* (KP) tables. Like other trivalent systems, the KP tables adopt the following convention:

#### Convention 1

Sentences denote one of the three values *“true”* (1), *“false”* (0), or, in cases of presupposition failure, *“undefined”* ($*$). When a sentence is true or false we say that its interpretation is *“well-defined”*.

Using this convention, a trivalent theory analyzes a bivalent proposition $\alpha$ as a presuppositional conclusion of a sentence *S* if $\alpha$ is true whenever *S* is “well-defined”, i.e., *true* or *false*. Equivalently, $\alpha$ is considered a presuppositional conclusion of *S* if it follows from *S* and its negation (van Fraassen, 1968).

As we saw in examples (8)–(10), incremental trivalent analyses like those of the KP tables correctly model the intuitive “filtering” of presuppositions with conditionals, conjunction and disjunction. However, the KP tables introduce “proviso problems” with these binary connectives. To see that, let us consider the following sentence: 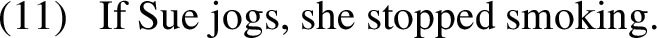


For (11) to be well-defined, the KP analysis requires one of two things: either Sue used to smoke or she does not jog. In the first case, the consequent is well-defined; hence, so is the full sentence. In the latter case, the antecedent is false, which makes (11) trivially true. Using the material implication treatment of conditionals, we conclude that (11) is well-defined in the KP analysis *if and only if* the following conditional statement holds: 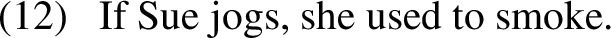


The ‘only if’ means that the KP tables expect (12) to be a presuppositional conclusion of (11). Furthermore, the ‘if’ means that any presuppositional conclusion of (11) logically follows from (12). In short, we say that the KP tables expect (12) to be the *logically strongest* presuppositional conclusion of sentence (11). This prediction fails to capture the fact that in out-of-the-blue contexts, hearers readily infer from (11) that Sue used to smoke, or, in current jargon, we say that they *project* the presuppositional content of (11)’s consequent (=‘Sue stopped smoking’).

Although the conditional (12) is not the logically strongest presuppositional conclusion of sentence (11), it intuitively supports felicitous utterances of this sentence. Suppose that hearers believe that Sue’s jogging is related to her smoking habits as stated in (12). Such hearers will not experience a presupposition failure in (11) even if they don’t know whether Sue actually used to smoke. Thus, (12) is an admittance condition of (11). Furthermore, we expect any situation where hearers accept (11) as felicitous to support (12). Thus, (12) is the logically *weakest* admittance condition of (11).

The KP tables capture this intuition. Using the KP tables, we define a *KP-admittance condition* of a sentence *S* as any bivalent proposition *C* such that the conjunction of *C* and *S* is well-defined under any interpretation. With this definition, the context (12) KP-admits (11), and any context *C* that KP-admits (11) entails (12).^4^ Thus, in agreement with intuition, the KP tables expect (12) to be (11)’s weakest admittance condition.

We have seen that the KP tables predict that the strongest presuppositional conclusion from sentence (12) is also (12)’s weakest admittance condition. This identification of the two notions is a general property of the KP tables, which is formally stated below:

#### Theorem 1

According to the KP analysis, the strongest presuppositional conclusion of a sentence *S* is equivalent to the weakest context that admits *S*.

This theorem is proved in using the formal analysis of KP-interpretations in .

Theorem 1 underlies the proviso problem for the KP analysis. In broader terms, this theorem is also relevant for dynamic accounts in possible world semantics. As Peters (1979) showed, the KP tables are descriptively equivalent to a possible-world interpretation of Karttunen’s (1974) proposal.^5^ The possible world analysis was further developed in much subsequent work on presupposition following Stalnaker (1973) and Karttunen (1974).^6^ The kind of congruence that Peters showed holds for these accounts as well. Thus, while there may be theoretical reasons to prefer possible world accounts to truth-functional accounts, for our present purposes it is sufficient to focus on the latter.

Incremental analyses like the KP tables and satisfaction-based methods often emphasize the (a)symmetric properties of presupposition projection.^7^ For conjunctions and conditionals, the asymmetry of the KP connectives is empirically welcome (Mandelkern et al., 2020). However, their strict incrementality introduces familiar challenges with disjunctions, as in the following examples (Roberts, 1989): 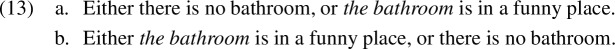


Neither sentence in (13) entails the existence of a bathroom. The KP tables capture this fact only in their analysis of (13a). Counterintuitively, the asymmetry of KP disjunction renders (13b) undefined in the absence of a bathroom. This is a reason to prefer the symmetric disjunction of the Strong Kleene tables. For the purposes of this paper, we adopt the asymmetric KP tables as the basis for developing the K-calculus. However, as we will demonstrate in Sect. 2.6, the same method allows us to define a “Karttunen-like” calculus corresponding to other truth-functional definitions. Consequently, the evaluation of (quasi-/a-)symmetric frameworks is orthogonal to our main proposal.

### Trivalent formulas and projection calculi

For studying presuppositions using trivalent truth tables, the informal analysis above is sufficient. However, in order to introduce the K-calculus and compare it to the KP tables, we formally define a propositional language that represents trivalent sentence meanings. We begin with a classical propositional language, denoted $$L_2$$, which is interpreted as bivalent. Standardly, $$L_2$$ formulas are either strings in some non-empty set *C* of elementary formulas (“constants”), or a combination of these constants using the classical operators $$\lnot $$, $$\wedge $$, $$\vee $$ and $$\rightarrow $$. Meanings of English sentences without propositional connectives are represented as pairs of such $$L_2$$ formulas: a presuppositional part and an assertive part. For example, sentence (14a) below is represented by the pair of bivalent formulas in (14b), where $$US $$ = ‘Sue used to smoke’ and $$S $$ = ‘Sue smokes’: 
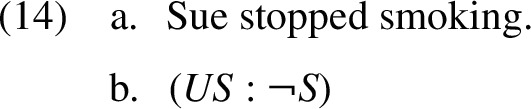


Intuitively, sentence (14a) is admissible if and only if Sue used to smoke. Under this condition, (14a) is equivalent to the statement ‘Sue does not smoke’. Accordingly, when the presuppositional part *US* is true, we interpret $(US : \lnot S )$ using the (bivalent) value of the assertion $\lnot S$. When *US* is false, $(US : \lnot S )$ is undefined.

Sentences containing propositional connectives are analyzed as propositional operations on pairs as in (14b). For example, the representation of sentence (11) (=‘if Sue jogs, she stopped smoking’) is as follows, where $$J $$=‘Sue jogs’: 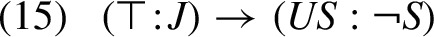


The presuppositional part of $(\top \!:\!J )$ is tautological ($\top$), as ‘Sue jogs’ is analyzed without any presuppositional import.

More generally, to represent trivalent propositions we use a propositional language, denoted $L_3$, which consists of pairs of $L_2$ formulas, as well as complex formulas constructed using propositional operators. Formally, we define:

#### Definition 2.1

*(trivalent language* $L_3$ *)* Given a propositional language $L_2$ over arbitrary constants, the *trivalent language* $L_3$ over $L_2$ is the closure of $L_2\times L_2$ under the propositional operators $\lnot$, $\wedge$, $\vee$ and $\rightarrow$.

Our goal is to systematically determine the presuppositional conclusions and admittance conditions of any given $L_3$ formula. We refer to such a mechanism as a *projection calculus*. For example, in a calculus that mimics the KP analysis of sentence (11) in Sect. 2.1, the bivalent statement $J \!\!\rightarrow \!\!US$ is derived as formula (15)’s strongest presuppositional conclusion, as well as its weakest admittance condition. In the following section, we introduce a projection calculus that corresponds to the KP tables in this way.

To represent admittance conditions in projection calculi, it is convenient to add a representation of contexts to our definition of $L_3$. Recall that when a sentence *S* is uttered in a context *C*, we represent this using the conjunction *C and S*. Using $L_3$ formulas, bivalent contexts like *C* should appear as $(\top \!:\!C)$, with a tautological presuppositional part. For example, when sentence (11) above (=‘if Sue jogs, she stopped smoking’) is uttered in the context of the sentence ‘Sue used to smoke’, we represent it in $L_3$ by conjoining $(\top \!:\!US )$ with the formula (15) as follows: 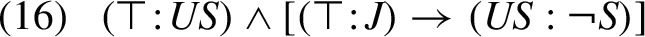 We abbreviate (16) by the following notation: 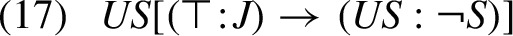


In general, we introduce the following notational convention:

#### Convention 2

For a trivalent formula $\kappa \in L_3$ *in the context of* a bivalent formula $\alpha \in L_2$, we use the notation:

$$\alpha [\kappa ]=(\top \!:\!\alpha )\wedge \kappa .$$

This shorthand, familiar from other satisfaction-based accounts, will be used freely hereafter.

### The Kleene–Peters (KP) calculus

Before presenting the K-calculus, we first examine the proof-theoretical counterpart of the KP tables, referred to as the *KP calculus*. This serves two objectives. First, the familiar KP tables help us to demonstrate the explicit analysis of projection and admittance in a logical calculus. Second, having a similar framework for describing the KP tables and the K-calculus facilitates the comparison of their semantic predictions. Since the KP semantics shares the implications of possible world accounts, this highlights the unique aspects of the K-calculus compared to both approaches.

We have seen that the semantics of the KP tables supports an informal analysis of projection and admittance. A projection calculus formalizes this analysis by mapping any trivalent formula $\kappa$ in $L_3$ to a bivalent formula ${\varvec{P}}(\kappa )$ in $L_2$. In the KP calculus, we aim for the derived proposition ${\varvec{P}}(\kappa )$ to accurately represent $\kappa$’s strongest presuppositional conclusion according to the KP tables. Furthermore, we expect the calculus to reflect KP-admittance of $\kappa$ by a context $\alpha$ by deriving a tautological statement as the strongest presuppositional conclusion ${\varvec{P}}(\alpha [\kappa ])$ for $\kappa$ within the context of $\alpha$.

To define the KP calculus on any trivalent formula $\kappa$, we will inductively employ the assertive contents of $\kappa$’s subformulas as well as their presuppositional contents. As for the inductive use of assertive contents, we first observe a simple fact: the assertive contents of any sentence with propositional connectives depend compositionally only on the assertive contents of its sub-parts and the bivalent semantics of its connectives. For instance, to know what the conditional (18a) below asserts we don’t need to think twice: it’s the conditional (18b) formed by the assertive parts of the two operands: 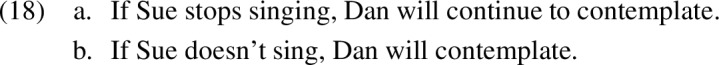


More generally, in Definition 2.2 below we formally introduce the *assertion operator* ***A*** over the trivalent language $L_3$:

#### Definition 2.2

*(assertive component)* The *assertive component* of any trivalent formula $\kappa \in L_3$ is the bivalent formula ${\varvec{A}}(\kappa )\in L_2$ that is inductively defined as follows:

$$ \begin{array}{lcl} {\varvec{A}}((\kappa _1\!:\!\kappa _2)) & = & \kappa _2 \\ {\varvec{A}}(\lnot \varphi ) & = & \lnot {\varvec{A}}(\varphi ) \\ {\varvec{A}}(\varphi \,{\textsf {op}}\,\psi ) & = & {\varvec{A}}(\varphi )\,{\textsf {op}}\,{\varvec{A}}(\psi ) \\ & & \text{ where } {\textsf {op}}\ \text{ is } \text{ any } \text{ binary } \text{ operator } \end{array} $$

Definition 2.2 specifies $\kappa$’s assertive component by inductively connecting the assertive contents $\beta$ of the elementary trivalent formulas $(\alpha \!:\!\beta )$ that make up $\kappa$. Importantly, this “assertion calculus” does not use any of the presuppositional contents (=the $\alpha$’s) within $\kappa$’s trivalent subformulas.^8^

The analysis of presupposition projection using the KP tables is more complex, as it requires us to consider both presuppositional and assertive components of sub-formulas. Specifically, in our discussion of sentences (8)–(10) above, we saw how the KP analysis requires checking whether the assertive content of the lefthand operand “determines” the result of the operation. To capture this idea formally, we define for any two-place bivalent operator ${\textsf {op}}$ a corresponding unary operator ${\textsc {ldv}}_{{\small {\textsf {op}}}}$. We refer to the ${\textsc {ldv}}_{{\small {\textsf {op}}}}$ operator as the *left-determinacy* operator associated with ${\textsf {op}}$. The ${\textsc {ldv}}_{{\small {\textsf {op}}}}$ operator sends any bivalent formula $\alpha$ to a formula that is true if and only if $\alpha$ has a truth-value that determines the bivalent value of the formula $\alpha \,{\textsf {op}}\,\beta$, independently of the value of $\beta$. For instance, when ${\textsf {op}}$ is material implication, we define:

$${\textsc {ldv}}_{\rightarrow }(\alpha )$$ is true

iff $\alpha$ determines the result of the implication $\alpha \!\!\rightarrow \!\!\beta$, for any bivalent $\beta$

Put differently, this means that the bivalent formula $(\alpha \!\!\rightarrow \!\!\top )\leftrightarrow (\alpha \!\!\rightarrow \!\!\bot )$ is true. More generally, we define for any binary operator *op* in a bivalent formula: 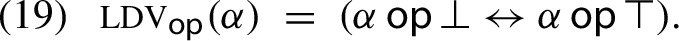


In words: a bivalent formula $\alpha$ has a left-determinant value of the operator ${\textsf {op}}$ if using $\alpha$ as the lefthand operand of ${\textsf {op}}$ makes the value of the operation indifferent to the value of its righthand operand. Consequently, for the bivalent binary connectives we get the following equivalences:
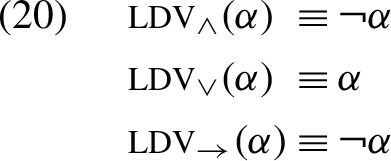


In words: $\alpha$ has an left-determinant value of conjunction (disjunction/implication) if and only if it is false (true/false, respectively).

The KP calculus uses the ${\textsc {ldv}}$ operator to map any trivalent formula $\kappa$ in $L_3$ to a bivalent formula ${\varvec{P}}^{\tiny {\text{ KP }}}(\kappa )$ in $L_2$. This is formally defined as follows:

#### Definition 2.3

*(KP calculus)* Let $\kappa \in L_3$ be a trivalent formula. We inductively define the bivalent formula ${\varvec{P}}^{\tiny {\text{ KP }}}(\kappa )\in L_2$ as follows:

$$\begin{aligned} \begin{array}{lcl} {\varvec{P}}^{\tiny {\text{ KP }}}((\kappa _1\!:\!\kappa _2)) & = & \kappa _1 \\ {\varvec{P}}^{\tiny {\text{ KP }}}(\lnot \varphi ) & = & {\varvec{P}}^{\tiny {\text{ KP }}}(\varphi ) \\ {\varvec{P}}^{\tiny {\text{ KP }}}(\varphi \,{\textsf {op}}\,\psi ) & = & {\varvec{P}}^{\tiny {\text{ KP }}}(\varphi )\wedge ({\varvec{P}}^{\tiny {\text{ KP }}}(\psi )\vee {\textsc {ldv}}_{{\small {\textsf {op}}}}({\varvec{A}}(\varphi ))) \end{array} \end{aligned}$$

To see how this definition treats the classical binary connectives, we can cash out its treatment using the equivalences in (20): 
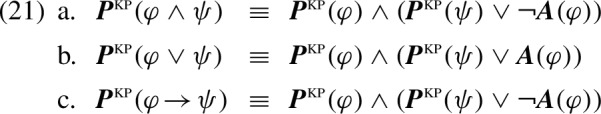


The next step is to verify that the KP calculus as defined above correctly mimics the operation of the KP tables. To establish that, we associate each complex trivalent formula $\kappa$ with the formula $({\varvec{P}}^{\tiny {\text{ KP }}}(\kappa )\!:\!{\varvec{A}}(\kappa ))$—the simple $L_3$ formula that consists of ${\varvec{P}}^{\tiny {\text{ KP }}}(\kappa )$ and $\kappa$’s assertive component. Provably, this simple trivalent formula is interpreted in the same way the KP tables interpret the original formula $\kappa$. In other words, we say that the KP calculus is *sound* with respect to KP-interpretations. Formally:

#### Fact 2.1

(soundness of KP calculus) For any trivalent formula $\kappa \in L_3$, the interpretation of $\kappa$ according to the KP tables equals the interpretation of the simple trivalent formula $({\varvec{P}}^{\tiny {\text{ KP }}}(\kappa )\!:\!{\varvec{A}}(\kappa ))$.

The proof of Fact 2.1 follows from Definition 2.3 by induction on the structure of $\kappa$ and the general definition of KP-interpretations in .

From Fact 2.1 it follows that for any trivalent formula $\kappa$, the formula ${\varvec{P}}^{\tiny {\text{ KP }}}(\kappa )$ derived by the KP calculus expresses the proposition that the KP tables model as $\kappa$’s strongest presuppositional conclusion. Let us illustrate this using sentences (8) and (11), reproduced below together with their $L_3$ representations: 
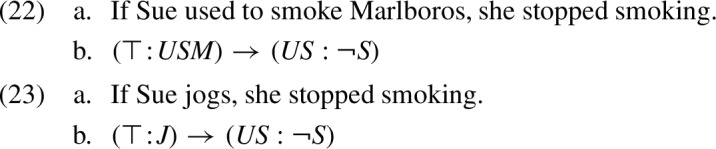


Both formulas (22b) and (23b) are of the form $(\top \!:\!\gamma ) \rightarrow (US : \lnot S )$. Applying the KP calculus to this formula leads to the following analysis:
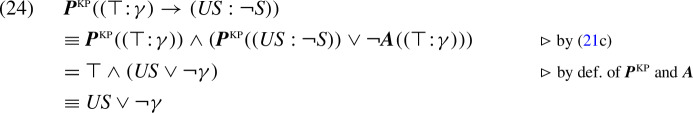


In example (22), we have $$\gamma =USM $$ (=‘Sue used to smoke Marlboros’). Thus, the KP calculus derives the formula $$US \vee \lnot USM $$. This is a tautology, as *USM* entails *US* (=‘Sue used to smoke’). We see that, in parallel to the analysis of “filtering” by the KP tables, and in agreement with intuition, sentence (22a) is treated as lacking any presupposition. By contrast, in (23) we have $$\gamma =J $$ (=‘Sue jogs’), which has no logical relation with $$US $$. Thus, like the KP tables, the KP calculus counterintuitively expects sentence (23) to have the presuppositional conclusion $$US \vee \lnot J $$, or, using material implication: ‘if Sue jogs, she used to smoke’.

We have seen that the soundness of the KP calculus corresponds with our informal analysis of presuppositional conclusions and admittance in Sect. 2.1. Formally, we state these alignments in the following corollary, which follows from the soundness of the calculus:

#### Corollary 1

For any trivalent formula $\kappa \in L_3$ and bivalent formula $\alpha \in L_2$:

- ${\varvec{P}}^{\tiny {\text{ KP }}}(\kappa )\Rightarrow \alpha$ iff $\alpha$ is a presuppositional conclusion of $\kappa$ according to the KP tables
- ${\varvec{P}}^{\tiny {\text{ KP }}}(\alpha [\kappa ])\equiv \top$ iff $\alpha$ admits $\kappa$ according to the KP tables

When the entailment ${\varvec{P}}^{\tiny {\text{ KP }}}(\kappa )\!\Rightarrow \!\alpha$ holds, we say in short that $\kappa$ *KP-presupposes* $\alpha$. When the equivalence ${\varvec{P}}^{\tiny {\text{ KP }}}(\alpha [\kappa ])\!\equiv \!\top$ holds, we say that $\alpha$ *KP-admits* $\kappa$. From Theorem 1, we conclude that $\kappa$’s strongest KP-presupposition, ${\varvec{P}}^{\tiny {\text{ KP }}}(\kappa )$, is also $\kappa$’s weakest KP-admittance condition. Formally:

#### Corollary 2

For any $\kappa \in L_3$: ${\varvec{P}}^{\tiny {\text{ KP }}}(\kappa )$ KP-admits $\kappa$, and is entailed by any $\alpha \in L_2$ that KP-admits $\kappa$.

This corollary will serve as a key point of comparison with the K-calculus in the next section.

### The Karttunen calculus (K-calculus)

In the previous sections, we have seen how, contrary to intuition, the KP analysis identifies presuppositional inference with admittance. This section introduces the Karttunen (*K*) calculus, whose incremental trivalent approach is similar to that of the KP tables. However, while the K-calculus generates the same admittance conditions as the KP tables, it yields stronger presuppositional inferences, in line with linguistic intuitions about projection.

Since Karttunen’s method relies on propositional contexts, we let the K-calculus manipulate items of the form $$\alpha [\kappa ]$$, as in Convention 2.^9^ The calculus is defined by mapping any item $$\alpha [\kappa ]$$ to a bivalent formula $${\varvec{P}}^{\tiny {\text{ K }}}(\alpha [\kappa ])$$, which we view as $$\kappa $$’s strongest presuppositional conclusion in the context of $$\alpha $$. When $$\alpha $$ is null, i.e., tautological, this represents a scenario where no prior assumptions are made; hence we view the result $${\varvec{P}}^{\tiny {\text{ K }}}(\top [\kappa ])$$ as $$\kappa $$’s *strongest presuppositional conclusion*, independently of context. For example, in a null context, sentence (23a) (=‘if Sue jogs, she stopped smoking’) is represented using the formula $$\top [(\top \!:\!J )\!\!\rightarrow \!\!(US \!:\!\lnot S )]$$. From this formula, the K-calculus derives the result $$US $$ (‘Sue used to smoke’), which adequately represents (23a)’s presuppositional conclusion. When we introduce the more specific context $$J \!\!\rightarrow \!\!US $$ (‘if Sue jogs, she used to smoke’), the K-calculus derives a tautological result, which correctly captures the intuitive admittance of sentence (23a) by this context.

Formally, we define the K-calculus inductively based on the structure of $\kappa$:

#### Definition 2.4

*(Karttunen calculus)* For any trivalent formula $\kappa \in L_3$ and bivalent context $\alpha \in L_2$, the bivalent formula ${\varvec{P}}^{\tiny {\text{ K }}}(\alpha [\kappa ])\in L_2$ is defined as follows:

$$\begin{aligned} \begin{array}{lcl} {\varvec{P}}^{\tiny {\text{ K }}}(\alpha [(\kappa _1\!:\!\kappa _2)]) & = & \left\{ \begin{array}{ll} \top & \alpha \Rightarrow \kappa _1 \\ \kappa _1 & \alpha \not \Rightarrow \kappa _1 \end{array} \right. \\ {\varvec{P}}^{\tiny {\text{ K }}}(\alpha [\lnot \varphi ]) & = & {\varvec{P}}^{\tiny {\text{ K }}}(\alpha [\varphi ]) \\ {\varvec{P}}^{\tiny {\text{ K }}}(\alpha [\varphi \,{\textsf {op}}\,\psi ]) & = & {\varvec{P}}^{\tiny {\text{ K }}}(\alpha [\varphi ])\,\wedge \,{\varvec{P}}^{\tiny {\text{ K }}}(\,\alpha '\,[\psi ]), \\ & & \text{ where } \ \alpha ' = \alpha \!\wedge \!{\varvec{P}}^{\tiny {\text{ K }}}(\alpha [\varphi ])\!\wedge \!\lnot {\textsc {ldv}}_{{\small {\textsf {op}}}}({\varvec{A}}(\varphi )) \end{array} \end{aligned}$$

In short, we refer to the formula ${\varvec{P}}^{\tiny {\text{ K }}}(\alpha [\kappa ])$ as $\kappa$*’s K-presupposition in* $\alpha$.

We can describe the three clauses in Definition 2.4 as follows:

- When $\kappa$ is a simple trivalent formula $(\kappa _1\!:\!\kappa _2)$, its K-presupposition in a context $\alpha$ is null (=tautological) if $\alpha$ entails (thus satisfies) $\kappa _1$, and it is $\kappa _1$ otherwise. As we will see, this is the main difference between the K-calculus and the KP analysis.
- Negation ($\kappa =\lnot \varphi$) is standardly defined as preserving K-presuppositions.
- When $\kappa$ is a binary construction $\varphi \,{\textsf {op}}\,\psi$, its K-presupposition in $\alpha$ is $\varphi$’s K-presupposition in $\alpha$, conjoined with $\psi$’s K-presupposition in a context $\alpha '$, which updates $\alpha$ using $\varphi$’s K-presupposition and assertive content. As we will see below, this is an adaptation of the incremental trivalent method.

Definition 2.4 generalizes Karttunen’s (1974) system. As in Karttunen (1974, p. 184), presuppositions of simple formulas are satisfied when they are logically entailed by their local context.^10^ Unlike Karttunen’s analysis, Definition 2.4 keeps $\kappa _1$ as the K-presupposition of $(\kappa _1\!:\!\kappa _2)$ if $\kappa _1$ is not entailed by the context. This is the core of the projection mechanism in the K-calculus, which will be useful in our analysis of presupposition accommodation in Sect. 3.

The treatment of a binary construction $\varphi \,{\textsf {op}}\,\psi$ is defined so that the context $\alpha '$ of the righthand operand $\psi$ “neutralizes” presuppositional effects of $\psi$ whenever $\varphi$’s assertive value left-determines the value of the operator ${\textsf {op}}$. This is obtained by negating the left-determinant value of $\varphi$ in relation to the ${\textsf {op}}$ operator. Thus, when $\varphi$ left-determines ${\textsf {op}}$, the context $\alpha '$ is false, which renders $\psi$’s K-presupposition in $\alpha '$ trivially true. As we will see in Sect. 2.6, this adjusts the treatment of binary connectives in the KP calculus to handle entailments between formulas in Karttunen’s proposal.

Let us illustrate the operation of the K-calculus in some simple examples. Satisfaction of sentence (25b) below in the context of (25a) is modeled by the tautological K-presupposition derived in (25c): 
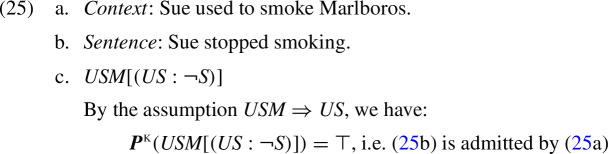


When the context in (25) is replaced by ‘Sue jogs’ (*J*), the sentence’s presuppositional part is not entailed by its context. Therefore, by Definition 2.4 we get: 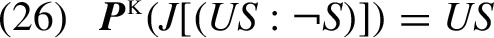


Thus, in the context of ‘Sue jogs’, the K-presupposition of sentence (25b) is that Sue used to smoke. The same K-presupposition is derived when (25b) is used in a null context.

The second clause in Definition 2.4 standardly preserves presuppositions under negation. For example, let us consider the K-presupposition of the negative sentence *Sue didn’t stop smoking* in the context ‘Sue jogs’: 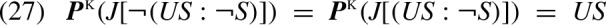


This is the same K-presupposition (26) as that of the positive sentence in (25b).

When it comes to binary connectives, Definition 2.4 utilizes the ${\textsc {ldv}}$ operator similarly to the KP calculus. Due to the equivalences in (20), we conclude: 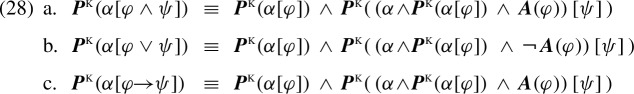


In words: in conjunctions and implications, the context of the second operand is obtained using the assertive part of the first operand; in disjunctions it is obtained using the negation of that assertive part. To exemplify this treatment, let us reconsider examples (22) and (23), reproduced below: 
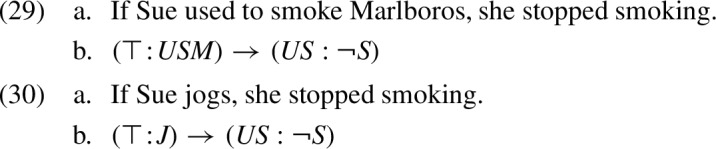


Both (29b) and (30b) are of the form $(\top \!:\!\gamma ) \rightarrow (US : \lnot S )$. In a null context $\top$, the treatment of implication in (28c) derives the following analysis for this formula: 
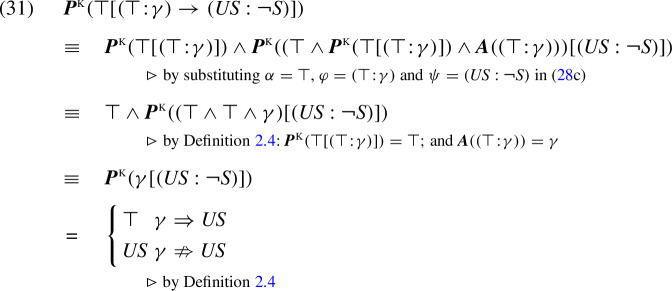


In example (29), we have $$\gamma =USM $$, which entails *US*. Thus, the result of analysis (31) is tautological. In (30), we have $$\gamma =J $$, which does not entail $$US $$, hence the result is $$US $$. In sum, we conclude: 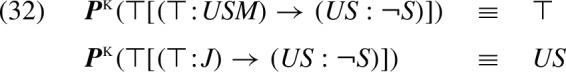


This accounts for the “filtering” effect in sentence (29a), as well as for the intuitive presuppositional conclusion from (30a). In contrast to the KP treatment of example (30) (in (23)), the K-calculus projects the presupposition of the consequent intact without unnecessarily “conditionalizing” it on the antecedent.

As we will see, despite this difference in presuppositional conclusions, admittance conditions in the K-calculus are the same as in the KP semantics. For example, let us reconsider sentence (30a), but now in the context of the conditional ‘Sue used to smoke if she jogs’. In this case, the K-calculus supports the following derivation: 
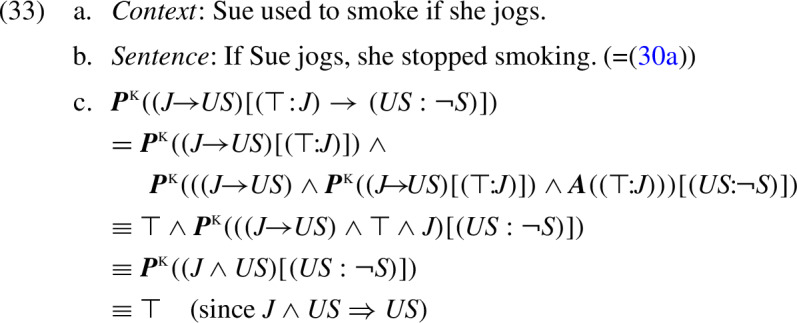


Thus, like the KP analysis, the K-calculus correctly treats (33a) as admitting (33b).

**Fig. 2:**
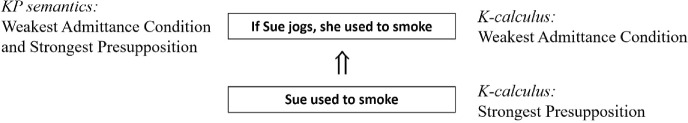
The weakest admittance condition and strongest presuppositional conclusion of sentence (33b) (=‘if Sue jogs, she stopped smoking’) in the K-calculus and in the KP calculus

The parallelism between the K-calculus and the KP system goes deeper than that. From the facts that will be shown below, we conclude that in the K-calculus, as in the KP semantics, (33a) is treated as the *logically weakest* admittance condition of (33b). Figure 2 summarizes our observations on the K-calculus and the KP calculus with respect to the conditional sentence (33b). In the following subsection, we formally establish the general differences and similarities between the two systems.

### Presuppositional conclusions vs. admittance in the K-calculus

We have seen that in the proposed K-calculus, a presuppositional conclusion from a sentence in a null context may be logically stronger than an admittance condition. This contrasts with the KP tables, where the strongest presuppositional conclusion coincides with the weakest admittance condition (Theorem 1). To compare the two systems more generally, we first formally define admittance within the K-calculus. Analogous to our definition of admittance using the KP tables, we say that a context *C* “K-admits” a sentence *S* when the conjunction of *C* and *S* has no unsatisfied K-presuppositions. Equivalently, the K-presupposition of *S* in *C* is tautological. Formally, we define:

#### Definition 2.5

*(K-admittance)* A bivalent formula $\alpha \in L_2$ *K-admits* a trivalent formula $\kappa \in L_3$ if ${\varvec{P}}^{\tiny {\text{ K }}}(\alpha [\kappa ])\equiv \top$.

Since we are often interested in a sentence’s K-presupposition in a null context, the following notation comes in handy:

#### Convention 3

For any trivalent formula $\kappa \in L_3$ we denote: ${\varvec{P}}^{\tiny {\text{ K }}}(\kappa )={\varvec{P}}^{\tiny {\text{ K }}}(\top [\kappa ])$.

The formula ${\varvec{P}}^{\tiny {\text{ K }}}(\kappa )$ is used for modeling $\kappa$’s strongest presuppositional conclusion independently of context. In short, we refer to it as $\kappa$*’s K-presupposition*.

With these notions of K-admittance and K-presupposition, we state two theorems that formally establish the general relations between the K-calculus and the KP tables. Theorem 2 below asserts that the admittance relation is identical in the K-calculus and the KP tables:

#### Theorem 2

For any bivalent formula $\alpha \in L_2$ and trivalent formula $\kappa \in L_3$:

$\alpha$ K-admits $\kappa$*iff*$\alpha$ KP-admits $\kappa$.

Due to this identity between K-admittance and KP-admittance, we henceforth use both terms interchangeably. Next, establishing a relation between admittance and presupposition in the K-calculus, Theorem 3 claims that the strongest presuppositional conclusion that the K-calculus derives for a sentence is sufficient for K-admitting it:

#### Theorem 3

For any trivalent formula $\kappa \in L_3$: ${\varvec{P}}^{\tiny {\text{ K }}}(\kappa )$ K-admits $\kappa$.

The proofs of Theorems 2 and 3 (Appendix B) follow by induction on the structure of trivalent formulas $\kappa \in L_3$.

By Theorem 1, the strongest KP-presupposition of a sentence is its logically weakest KP-admittance condition. Thus, from Theorems 2 and 3 we infer the following logical relations between K-presupposition and K-admittance, or, equivalently, between K-presupposition and KP-presupposition/admittance:

#### Corollary 3

For any trivalent formula $\kappa \in L_3$: ${\varvec{P}}^{\tiny {\text{ K }}}(\kappa ) \Rightarrow {\varvec{P}}^{\tiny {\text{ KP }}}(\kappa )$.

Importantly, there is no entailment in the opposite direction: K-presuppositions may be properly stronger than K/KP-admittance conditions, as we saw in the analysis of example (30) above.

**Fig. 3:**
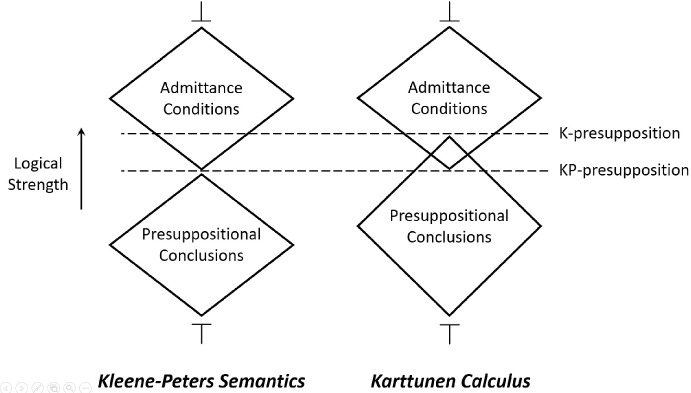
The derived K-presupposition of a sentence may be logically stronger than its strongest KP-presupposition. By contrast, weakest admittance conditions are the same in both systems, and equivalent to this KP-presupposition. Tautology ($\top$) is the trivial presuppositional conclusion (=follows from any sentence), whereas contradiction ($\bot$) is the trivial admittance condition (=admits any sentence)

These meta-theoretical conclusions are summarized in Fig. 3.

### On symmetric projection and general K-systems

**Fig. 4:**
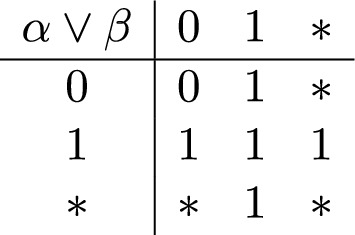
Strong Kleene disjunction


Section 2.1 mentioned symmetric presupposition projection with disjunctions as in sentences (13a-b), repeated below: 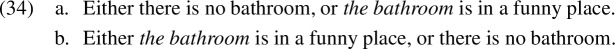


Like the KP tables, the K-calculus accounts for left-to-right filtering as in (34a), but not for the right-to-left filtering in (34b). In this section, we show that any trivalent Kleene-like table can be transformed to a parallel Karttunen-like calculus. In particular, this holds for symmetric disjunction. To illustrate, consider replacing the asymmetric KP disjunction by the symmetric Strong Kleene table in Fig. 4. This motivates the replacement of the asymmetric disjunction rule (35) in the KP calculus with the symmetric rule for disjunction in (36):

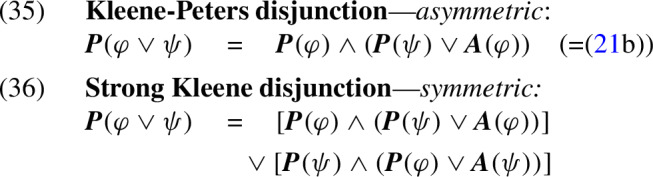


Similarly, to treat symmetric filtering as in (34), we can replace the disjunction rule in the K-calculus (28b), restated in (37), by the revised symmetric recipe in (38) (cf. Karttunen 1974, p. 185): 
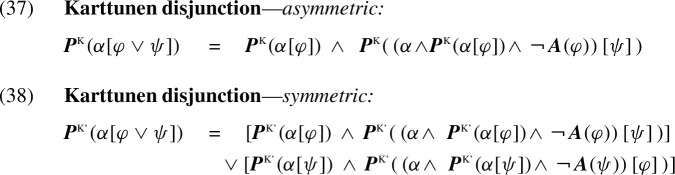


In (38), the second disjunct adds $\psi$ to the context of evaluating the presupposition of $\varphi$. This modification introduces symmetry into the rule, treating (34b) similarly to the analysis of (34a) in the K-calculus.

This emulation of symmetric truth tables using a K-like calculus raises a more general question: how is the K-calculus or variations thereof related to calculi that model trivalent truth tables? The answer is surprisingly simple: the two kinds of calculi only differ in their rules for *simple* formulas. To exemplify that, let us consider the sentence *Sue stopped smoking* in the context of the statement ‘Sue jogs’. The analyses of this situation in the KP and K calculi are given in (39a) and (39b) (=(26)) below, respectively:
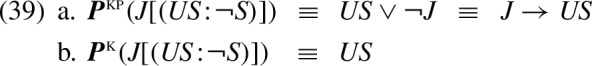


This is in a nutshell the “proviso” difference between the KP semantics and the K-calculus. In the K-calculus, only lexical K-presuppositions (e.g., *US* in (39b)) that are not entailed by their local context are projected. By contrast, the KP calculus can result in modification of lexical presuppositions. Thus, it also generates formulas like $J \!\!\rightarrow \!\!US$ in (39a), which is not lexically triggered.

In all other respects, the projection rules of the two calculi are fully aligned. However, this claim is not immediately obvious in the case of binary operations. To clarify, let us observe the following fact about the KP calculus (our reason for underlining part of the equation will become clear presently):

#### Fact 2.2

For all trivalent formulas $\varphi ,\psi$ in $L_3$:

$$ {\varvec{P}}^{\tiny {\text{ KP }}}(\varphi \,{\textsf {op}}\,\psi ) \equiv {\varvec{P}}^{\tiny {\text{ KP }}}(\varphi )\,\wedge \,{\varvec{P}}^{\tiny {\text{ KP }}}((\underline{{\varvec{P}}^{\tiny {\text{ KP }}}(\varphi )}\!\wedge \!\lnot {\textsc {ldv}}_{{\small {\textsf {op}}}}({\varvec{A}}(\varphi )))[\psi ])$$

$$This \, equivalence \, mirrors \, the \, binary \, construction \, rule \, in \, K-calculus \, (Definition $$ 2.4):

$${\varvec{P}}^{\tiny {\text{ K }}}(\varphi \,{\textsf {op}}\,\psi ) = {\varvec{P}}^{\tiny {\text{ K }}}(\varphi )\,\wedge \,{\varvec{P}}^{\tiny {\text{ K }}}((\underline{{\varvec{P}}^{\tiny {\text{ K }}}(\varphi )}\!\wedge \!\lnot {\textsc {ldv}}_{{\small {\textsf {op}}}}({\varvec{A}}(\varphi )))[\psi ])$$

Fact 2.2 is proved in . It demonstrates an equivalent formulation of the rule for binary operations in the KP calculus, which parallels the rule of the K-calculus, leaving the satisfaction rule of simple formulas the only difference between the two calculi. Similarly, the symmetric disjunction rules (36) and (38) can be demonstrated to correspond.

We have seen that the K-calculus can be modified to incorporate symmetric projection rules, consistent with the treatment of (a)symmetry within trivalent semantics. Using a parallel method, we can transform any trivalent projection calculus $\mathcal{C}$ into a K-variant $\mathcal{C^\textrm{K}}$. In the case of the KP calculus and the K-calculus, we saw in Sect. 2.5 that the K-presupposition of a sentence may be logically stronger than its strongest KP-presupposition, although the weakest admittance conditions remain equivalent in both systems. We hypothesize that this relationship holds more generally, as stated in the following conjecture:^11^

#### Conjecture 1

Let $\mathcal{C}$ be a projection calculus based on trivalent truth tables, with a parallel K-variant $\mathcal{C^\textrm{K}}$. The $\mathcal{C}^K$-presupposition of any trivalent formula is logically stronger than, or equivalent to, its $\mathcal{C}$-presupposition. By contrast, the admittance conditions of any formula are equivalent in the two calculi.

If correct, this conjecture could prove valuable in allowing us to rely on general properties of Karttunen’s entailment-based satisfaction for trivalent systems where projection from binary constructions is symmetric (as in Kleene’s systems), asymmetric (as in the KP system) or mixed (e.g., symmetric for disjunction and asymmetric for other operations). Further investigation of such systems is left for further research.

*Note on the definition of local contexts*. The treatment of local contexts in the K-calculus warrants some further explanation. From Fact 2.2, it follows that introducing the underlined term ${\varvec{P}}^{\tiny {\text{ KP }}}(\varphi )$ within the KP system is innocuous and thus unnecessary. Why, then, did we introduce the corresponding underlined term ${\varvec{P}}^{\tiny {\text{ K }}}(\varphi )$ in the K-calculus? The reason is that omitting this clause would yield a K-like system that is empirically inadequate compared to the K-calculus, and also inferior to the KP semantics. To illustrate this, let us consider the following example: 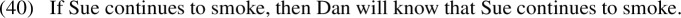


From (40), we infer the presuppositional conclusion that Sue used to smoke, which is simply accounted for as a projection of the antecedent’s presupposition. Importantly, however, sentence (40) does not entail that Sue still smokes—in a null context, it may be admitted even if Sue used to smoke but has since quit. Thus, one part (‘Sue used to smoke’) of the factive’s presupposition is filtered, while another part (‘Sue smokes now’) is projected from the antecedent.^12^ In the K-calculus, presupposition filtering is a matter of all-or-nothing. For example, in (40), the assertive part (‘Sue still smokes’) of the antecedent does not entail the factive’s presupposition (‘Sue used to smoke and still does’). We conclude that in the K-calculus, the presupposition ‘Sue used to smoke’ of (40)’s antecedent must also be involved in filtering the presupposition of the consequent. This is our motivation for introducing the K-presupposition ${\varvec{P}}^{\tiny {\text{ K }}}(\varphi )$ of the antecedent into the context of the consequent (underlined in Fact 2.2 above).

To verify that the resulting K-calculus rule functions as intended, let us consider the derivation for sentence (40) in (42), using the notation in (41):
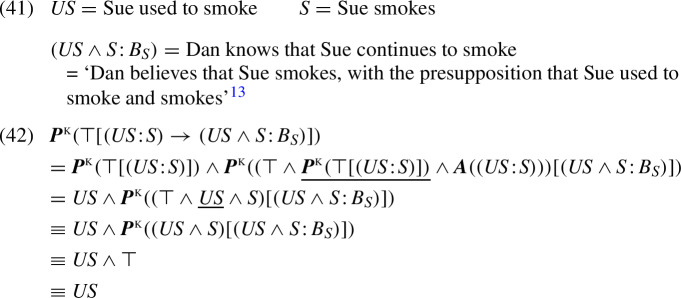
13

The underlined part in (42) highlights the presupposition $${\varvec{P}}^{\tiny {\text{ K }}}(\top [(US \!:\!S )])$$ from (40)’s antecedent (‘Sue continues to smoke’) as part of the local context for the consequent $$(US \wedge S \!:\!B_{{\tiny {S }}})$$ (‘Dan knows that Sue continues to smoke’). Together with this presupposition (=$$US $$, ‘Sue used to smoke’), the antecedent filters the consequent’s presupposition $$US \wedge S $$ (‘Sue used to smoke and smokes’). This example provides further justification for the definition of binary operations in the K-calculus.

## Pragmatic accommodation with the $\text{ Karttunen }$ calculus

The previous section introduced the K-calculus and the distinction it draws between presuppositional conclusions and admittance conditions. The logically strongest presuppositional conclusion of a sentence is modeled by the *K-presupposition* derived by the K-calculus in a null context. By contrast, a *K-admittance* condition is defined as any context that renders a sentence’s presuppositional conclusion tautological.

Like other semantic approaches to presupposition, the K-calculus does not on its own account for presupposition accommodation: the pragmatic process by which hearers incorporate presupposed content into their representation of the common ground during a conversation. This leaves certain aspects of the “proviso problem” unaddressed. The challenge for the K-calculus is that hearers sometimes accommodate information that is logically weaker than, or independent of, a sentence’s K-presupposition. Following Karttunen (1973, 1974), we articulate this pragmatic strategy, building on further observations by Geurts (1996) and Mandelkern (2016a, 2016b). In the proposed *K-accommodation* strategy, the K-presupposition of an utterance is the first candidate that a hearer considers for accommodation when a sentence’s admittance conditions are not satisfied. However, if another statement that satisfies the admittance conditions is pragmatically more plausible than the K-presupposition, the hearer will prefer accommodating that statement. This approach contrasts with standard analyses like the KP tables or dynamic possible world semantics, where the sentence’s weakest admittance condition (=standard ‘presupposition’) is the default for accommodation. Empirical differences between these methods emerge when there is no pragmatic pressure favoring an alternative to the semantically derived presupposition. Following Geurts and Mandalkern—and ultimately Karttunen—we argue that in such cases, speakers prefer to accommodate the K-presupposition rather than the standard presupposition. This preference highlights the advantage of the K-calculus over previous semantic accounts that do not distinguish presuppositions from admittance conditions.

### Accommodation: strengthening presuppositions or defeating them?

Let us first consider the following example from Katzir and Singh (2013):^14^
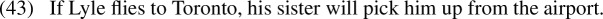


When hearing (43) out of the blue, we reasonably infer that Lyle has a sister. Thus, the proposition that we consider as the semantic presuppositional conclusion from (43) is also pragmatically inferred. How does this inference work? To analyze this, let us first review some familiar pragmatic notions from (Stalnaker, 1974). When hearers interpret a sentence, they do that while assuming a proposition *C*, which they consider the *common ground* of the conversation. Adopting Stalnaker’s (1974/1999, p. 49) notion of “pragmatic presupposition”, we can intuitively describe the common ground as follows: 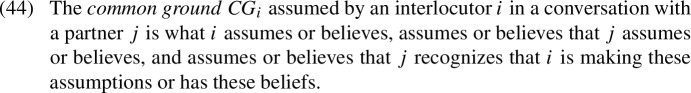


Ideally, the common ground *C* that hearers assume (=$CG _H$) admits any sentence *S* that they hear. In such cases, *C* is updated by *S*’s assertive content and the conversation goes on as smoothly as possible. However, actual exchanges of information do not always go in this ideal way. In practice, hearers may often encounter sentences that are not admitted by the common ground they have previously assumed. In such cases, cooperative interlocutors can adjust their assumptions to maintain the conversation. For example, upon hearing the utterance *I’ve got to pick up my sister*, a hearer unaware that the speaker has a sister might naturally accommodate this information without further questions (Stalnaker 1974/1999, p. 52). In general terms, we describe this kind of pragmatic inference as follows: 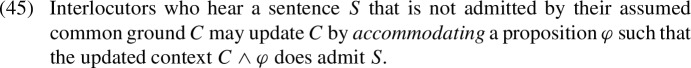


In general, hearers interpreting a sentence *S* in a common ground *C* typically have three options. If *S* is admitted by *C*, they immediately update *C* using *S*’s assertive content (i). If *C* does not admit *S*, hearers can accommodate some $\varphi$ such that $C\wedge \varphi$ admits *S* (ii). Otherwise, hearers can ask for clarifications (iii). The third reaction typically occurs when hearers do not hold the relevant assumptions, but believe they should have known them if they were true. For instance, this might be the reaction of a number theorist who is being told that *the mathematician who proved Goldbach’s Conjecture is from Yale* (von Fintel, 2004, 2008).

Now let us get back to sentence (43). When uttered in a null context, semantic approaches like KP semantics or the Heim-Stalnaker account derive the following conditional as (43)’s unitary semantic “presupposition”: 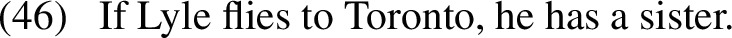


This conditional is weaker than the inference hearers usually draw: 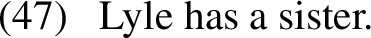


Standard accounts address this problem by pragmatically *strengthening* (46) into (47). According to this analysis, the unlikelihood of the connection that (46) makes between flying to Toronto and having a sister leads hearers to replace (46) by (47), which is then accommodated into their assumed common ground $CG _H$.^15^

In our pragmatic account using the K-calculus, we take the K-presupposition as the first candidate for accommodation. Out of the blue, when the context $CG _H$ is informationally null (=tautological), the K-calculus analyzes sentence (43) with (47) as its K-presupposition. In this case there is no reason to accommodate any weaker admittance condition, especially not the weakest K-admittance condition (46), which is pragmatically odd. Accordingly, the K-calculus directly accounts for the observed inference of (47) from (43).

The situation is different with examples like the following:^16^
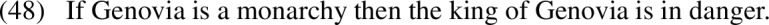


Out of the blue, we do not infer from (48) that Genovia has a king. Standard accounts analyze this sentence with the presupposition: 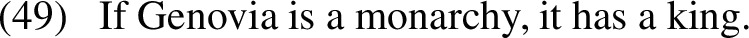


The connection that (49) makes between monarchies and kings is perfectly coherent. Accordingly, standard accounts expect (49) to be accommodated upon hearing (48) without any strengthening.

Using the K-calculus in a null context, the following K-presupposition is projected intact from (48)’s consequent: 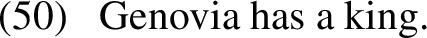


This is so because (48)’s antecedent does not entail having a king: a monarchy could plausibly have a ruler who is not a male. Therefore, using the K-calculus, we need to explain why hearers, upon hearing (48) out of the blue, do not directly accommodate (50). The reasoning here diverges from the strengthening analysis.^17^ Accommodating (50) is problematic because this statement entails the antecedent of (49) (=*Genovia is a monarchy*). Thus, if the speaker had (50) in the assumed *CG*, that would violate Grice’s (1975) ignorance implicature about conditionals. Accordingly, the hearer is motivated to search for alternative candidates for accommodation, i.e., other propositions that entail (48)’s K-admittance condition (49). One plausible alternative is the following generic statement: 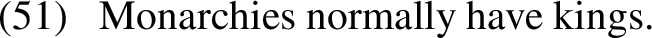


Given the history of monarchies, (51) is a fairly natural assumption. Furthermore, in the lack of shared knowledge about Genovia, both hearer and speaker are likely to assume the following conditional pattern: 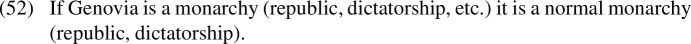


From these default assumptions it follows that (50) holds.^18^ Thus, if the speaker has (51) in the assumed *CG* without specific details about Genovia, the K-calculus correctly expects sentence (48) to be admitted. In such a null context, where the speaker is aware that her hearers know nothing about Genovia, she is more likely to assume the generic sentence (51) than to assume the Genovia-specific claim in (50). As a result, in typical conversations, hearers are expected to favor (51) over (50) as their candidate for accommodation.

### K-accommodation

The pragmatic alternative we propose using the K-calculus begins with the K-presupposition as the initial candidate for accommodation, but replaces it by another candidate that K-admits the sentence if there is a pragmatic reason to do so. We refer to this strategy as *K-accommodation*. At first glance, K-accommodation may look like the mirror image of the strengthening approach. However, the pragmatic assumptions of the two approaches are notably different. The following example from Geurts (1996) nicely illustrates one such difference: 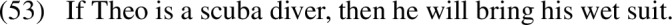


Standard semantic approaches derive for (53) the following presupposition: 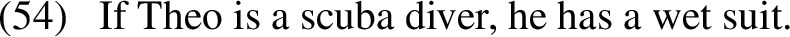


Strengthening is not necessary in this case, since (54) is a perfectly coherent statement. Consequently, standard approaches treat (54) as the only candidate for accommodation when (53) is heard in a null context. By contrast, the K-presupposition of (53) in a null context is: 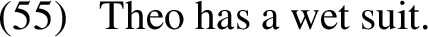


Empirically, speakers do not consistently infer (55) when they encounter (53). In contrast to example (48), discussed above, there is nothing pragmatically deviant in suggesting that the speaker has included (55) in her postulated *CG*. The challenge for K-accommodation is then: why isn’t (55) consistently inferred from (53)? The explanation proposed here is that the following generic statement is substantially more plausible than (55) to be part of the speaker’s assumed *CG*: 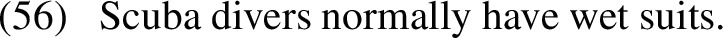


If (56) is assumed in the *CG*, then the lack of specific information about Theo entails the conditional (54), similarly to our analysis of (48) above. Thus, given that the speaker is aware that the hearer *H* knows nothing about Theo, it is more reasonable to accommodate a generic statement like (56) into *H*’s assumed *CG* rather than the Theo-specific assertion (55). It is important to observe that this reasoning does not apply in the case of (43). Unlike the scenario in (53), in (43) it would be odd for hearers to consider the generic statement *fliers to Toronto normally have sisters* as a more likely part of the speaker’s *CG* than (47).

In general, we define K-accommodation as follows. To interpret an utterance of a sentence *S* against an assumed common ground $C=CG _H$, a hearer *H* uses the following set of propositions based on *H*’s estimations regarding the speaker’s common ground $CG _S$: 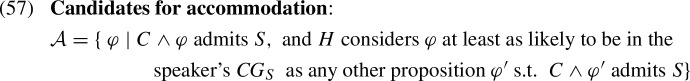


In K-accommodation, the hearer starts with the K-presupposition *p* of *S* in *C* as a basis, but disregards it if it not found among the likeliest candidates in $\mathcal{A}$.^19^ Formally: 
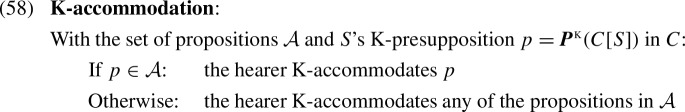


According the K-accommodation strategy, the hearer may always accommodate a statement $\varphi$ that the speaker is likelier to assume than the K-presupposition, as long as $C\wedge \varphi$ admits the speaker’s utterance. This process explains why (54) is pragmatically inferred from (53).

K-accommodation also captures a phenomenon commonly referred to as “semi-conditional” presuppositions (Geurts, 1996; Singh, 2007; Schlenker, 2011). This concerns variants of (53), such as the following: 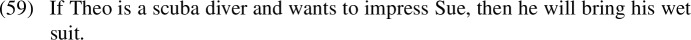


Geurts observes that from (59), as with (53), hearers naturally draw the conditional inference (54), which is logically stronger than (59)’s admittance condition below: 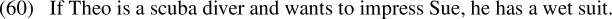


As with (53), the K-presupposition of sentence (59) in a null context is *Theo has a wet suit* (=(55)). K-accommodation analyzes (59) by assessing the plausibility of this K-presupposition against other propositions that admit (60). In particular, a hearer may consider the following generic sentences: 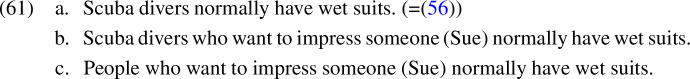


Among these statements, only (61a) may reasonably be considered as substantially more plausible than (55) to be part of the common ground. As a result, (61a) is K-accommodated rather than (55) or alternative assumptions like (61b) and (61c).

### Comparing K-accommodation to pragmatic strengthening

K-accommodation differs from more standard approaches in taking the K-presupposition to be the default inference: all else being equal, the hearer will accommodate the K-presupposition and disregard other propositions that make the context admit the utterance. By contrast, in more standard approaches, the first candidate for accommodation is the weakest admittance condition. One type of empirical difference between the two approaches is illustrated in the following example by Mandelkern (2016b):^20^
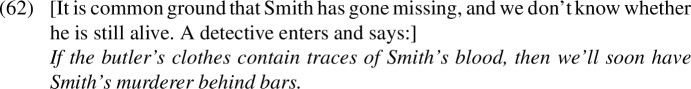


The conclusion that hearers are likely to draw from (62) is that Smith was murdered. This poses a challenge for standard approaches, but aligns with the predictions of K-accommodation. To see why this is the case, consider the K-presupposition and the weakest K-admittance condition of (62), which are as follows: 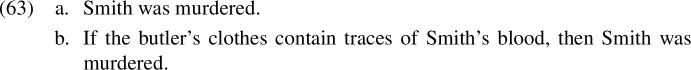


The dependence that (63b) creates between finding traces of Smith’s blood and Smith’s murder is entirely plausible and seems as likely as (63a) to be part of the detective’s assumed common ground. As Mandelkern observes, this implies that standard pragmatic approaches incorrectly predict that hearers would accommodate (63b) rather than (63a). In contrast, for K-accommodation to fail in a similar manner, hearers would need to estimate that (63b), or perhaps the following generic statement, is significantly more plausible as part of the common ground than (63a): 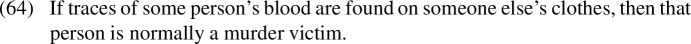


However, neither (64) nor (63b) appears to be pragmatically more plausible than (63a). According to K-accommodation, this implies that hearers have no reason to dismiss the K-presupposition (63a). Consequently, they are expected to K-accommodate it, which is consistent with the observed inference.

Another instance where K-accommodation proves advantageous over more standard pragmatic approaches arises in contrasts such as the following (Geurts, 1996, p. 278): 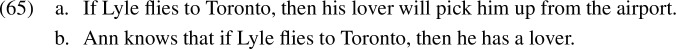


From sentence (65a), similarly to (43), we intuitively infer: 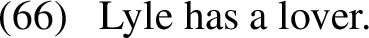


By contrast, from sentence (65b) we intuitively infer the following conditional: 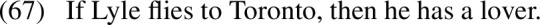


Standard analyses predict that (67) serves as the unitary presupposition for both (65a) and (65b). In the case of (65a), this prediction is analogous to the analysis of (43) above. Regarding (65b), the factive verb *know* directly triggers the conditional presupposition (67), which is subsequently projected as the presupposition of (65b). The question is: do standard analyses expect hearers to strengthen (67) into (66)? This question is not easily resolved on general pragmatic grounds, but importantly, either resolution poses challenges to the standard approach. Strengthening (67) to (66) yields counterintuitive results in (65b), while leaving (67) unstrengthened creates problems with treating (65a).

K-accommodation avoids this dilemma. While (65a)’s K-presupposition is $p_1$=(66), sentence (65b) K-presupposes the conditional $p_2$=(67). Both $p_1$ and $p_2$ seem to be equally plausible as part of the assumed common ground. This predicts that the speaker could have reasonably assumed either proposition. As a result, the hearer has no reason to reject the K-presupposition in either case. The outcome is intuitive enough: in (65a), hearers will K-accommodate $p_1$, while in (65b), they will K-accommodate $p_2$, diverging from the predictions of standard presupposition strengthening.

### Loose ends in the analysis of accommodation

We have proposed that upon hearing a sentence *S*, hearers by default accommodate *S*’s K-presupposition. Other statements that admit *S* are only accommodated when hearers have a reason to believe they are likelier to be assumed by the speaker than the K-presupposition. This notion of “likelihood” is part of all accounts of the proviso problem (Fox, 2013, pp. 222–223), but is not easy to define. To make theories of accommodation more predictive, one way is to enrich a probabilistic theory as in Lassiter (2012) with an explanatory account of probabilistic (in)dependence between statements. Lassiter (2012, pp. 17-19) postulates that conditionals as in (54) (=‘if he’s a diver, he has a wetsuit’) are *a priori* plausible because of the dependency between the antecedent and the consequent, whereas such a dependency does not exist in conditionals like (67) (=‘if he flies to Toronto, he has a lover’). Although this is intuitive enough, we should like to account for these judgments using general principles. Such principles are not part of theories of accommodation (including Lassiter’s), which aim to account for effects of world knowledge on presupposition, but not for the principles that govern this knowledge. This reduces the predictive power of all theories of accommodation. However, also without a principled account of “likelihood”, intuitions about this notion can be tested empirically, for instance by comparing speaker judgments on generics like (51) (=‘monarchies have kings’) and (56) (‘divers have wetsuits’) as opposed to sentences like the following: 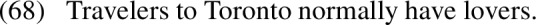


We expect a correlation between acceptance of generics like (51), (56) and (68) and accommodation of conditional inferences in cases like (48), (53) and (65a), respectively. Establishing such correlations might also allow us to test subtle differences between theories of accommodation. This is a major experimental effort that has yet to be undertaken.

Another factor that affects the evaluation of theories of accommodation involves coherence effects that are not necessarily related to accommodation *per se*. To see that, let us consider the following example:^21^
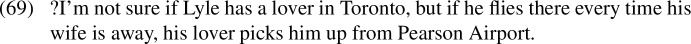


In (69), the speaker openly expresses uncertainty about whether Lyle has a lover. By zeroing out the likelihood that the speaker assumes this K-presupposition, this leads the K-accommodation strategy to expect that hearers accommodate another proposition that admits (69), e.g., a logically weaker conditional like (67). Why doesn’t (69) nevertheless sound coherent? We might consider this as a counterexample to the K-accommodation strategy, but it may also stem from independent factors of sentence coherence. First, let us note that putting the focus on *his lover* as in (70) below improves (69) considerably: 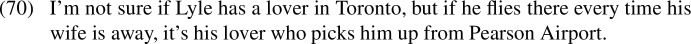


Second, similar incoherence effects to (69) appear even in cases where K-accommodation does expect conditional inferences. For instance: 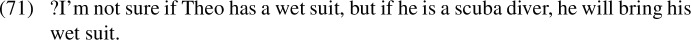


These facts suggest that the incoherence of (69) is connected to general focus principles and not to accommodation of presuppositional material *per se*. Similar (in)coherence effects also arise when no presuppositions are involved, as in the following examples: 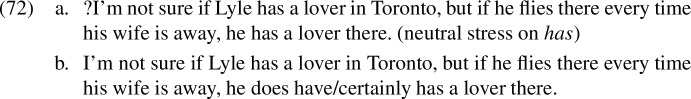


Stressing the second *has* in (72) improves the coherence of the assertion, similar to the (in)coherence effects with the presupposition in (69) and (70) above. This suggests that presupposition accommodation is sensitive to similar principles about information structure as direct assertion. We conclude that a pragmatic filter of sentence coherence must be superimposed on any theory of accommodation. Like the question of likelihood of generic propositions and their effects on the theory of accommodation, this point is at least partly orthogonal to the accommodation mechanism, and its elaboration is left for further research.

## Conclusion

We distinguished two logical notions central to formal semantics and pragmatics: presuppositional conclusions, defined as inferences with exceptional projection properties; and admittance conditions, which reflect the Strawsonian intuition that sentences may resist truth-value judgments in specific contexts. We observed that semantic theories of presuppositions naturally connect these notions. A sentence is admitted in a given context if the only presuppositional conclusion from their conjunction is tautological. We examined two such theories: the Kleene-Peters trivalent truth tables and Karttunen’s dynamic approach. These theories were given a general formalization as calculi for presuppositional projection with trivalent formulas, providing a basis for comparing their logical properties. It was found that although the KP calculus and the K-calculus agree on admittance conditions and share the principle of value determination, they diverge on how presuppositional conclusions are derived. Karttunen’s entailment-based treatment operates on logical forms, enabling unsatisfied admittance conditions to project. The emerging distinction between the logical strength of projected inferences and admittance conditions informs our proposed solution to the “proviso problem”. In our pragmatic proposal, presuppositional conclusions are treated as primary candidates for accommodation, while allowing pragmatically more plausible admittance conditions to take precedence. This integration of projection and admittance into formal semantics and pragmatics clarifies the analysis of presupposition and explains the prominence of lexical presuppositions in pragmatic reasoning. Furthermore, the proposed framework suggests a promising direction for advancing experimental research on accommodation in discourse and enriching philosophical discussions on the semantics-pragmatics interface.

## Kleene–Peters (KP) interpretations

This appendix defines the interpretation of the trivalent language $L_3$ using the KP tables, which allows us to establish Theorem 1 in Appendix B. A bivalent interpretation $[ \! [ \cdot ] \! ]^{{ bi}}$ of propositional $L_2$ formulas is routinely obtained by extending an arbitrary interpretation of the propositional constants in $L_2$ using the standard bivalent truth tables. Simple trivalent formulas in $L_3$, i.e. pairs of $L_2$ formulas, are interpreted using Blamey ’s (1986) *transplication* operator, a basic version of which is defined below:

### Definition A.1

*(transplication)* Given a bivalent interpretation $[ \! [ \cdot ] \! ]^{{ bi}}$ of $L_2$, for any $\alpha ,\beta \in L_2$, we define the trivalent interpretation $[ \! [ (\alpha \!:\!\beta ) ] \! ]^{{ bi'}}$ as follows:

$$\begin{aligned} [ \! [ (\alpha \!:\!\beta ) ] \! ]^{{ bi'}} = \left\{ \begin{array}{ll} [ \! [ \beta ] \! ]^{{ bi}} & [ \! [ \alpha ] \! ]^{{ bi}}=1 \\ *& [ \! [ \alpha ] \! ]^{{ bi}}=0 \end{array} \right. \end{aligned}$$

In words: when the basic presupposition $\alpha$ is true, the pair $(\alpha \!:\!\beta )$ is evaluated like the bivalent assertive content $\beta$; when $\alpha$ is false, $(\alpha \!:\!\beta )$ is “undefined”.

Complex formulas in $L_3$ are interpreted using the KP tables, which are based on the notion of *left-determinant value* (cf. George, 2014). Formally, we define:

### Definition A.2

*(KP binary operators)* Let ${\textsf {op}}$ be a binary operator with the bivalent truth table $f\!:\!(\{0,1\}\!\times \!\{0,1\})\!\rightarrow \!\{0,1\}$. The trivalent *KP truth table* of ${\textsf {op}}$ is the function $[ \! [ {\textsf {op}} ] \! ]^{\tiny { KP}}\!:\!(\{0,1,*\}\!\times \!\{0,1,*\})\!\rightarrow \!\{0,1,*\}$ that for any $u,v\in \{0,1,*\}$ is defined by:

$$\begin{aligned} [ \! [ {\textsf {op}} ] \! ]^{\tiny { KP}}(u,v) \!=\! \left\{ \begin{array}{lll} f(u,v) & u,v\!\in \!\{0,1\} \\ f(u,1) & u\!\in \!\{0,1\} \text{ and } f(u,1)\!=\!f(u,0), \text{ i.e., } u \\ & \text{ is } \text{ a } \text{ left-determinant } \text{ value } \\ *& \text{ otherwise } \end{array} \right. \end{aligned}$$

A KP-interpretation of a formula in $L_3$ inductively uses standard trivalent negation with this KP semantics of binary operators, together with the transplication-based interpretation of simple trivalent components:

### Definition A.3

*(KP-interpretation of* $L_3$ *)* Let $[ \! [ \cdot ] \! ]^{{ bi}}$ be a bivalent interpretation of $L_2$, and let $[ \! [ \cdot ] \! ]^{{ bi'}}$ be the corresponding interpretation of $L_2\times L_2$ (Definition A.1). For any trivalent formula $\kappa \in L_3$, the *KP-interpretation* of $\kappa$ is denoted $[ \! [ \kappa ] \! ]^{\tiny { KP}}$ and is defined inductively as follows:

$$\begin{aligned} [ \! [ (\kappa _1\!:\!\kappa _2) ] \! ]^{\tiny { KP}}= &  [ \! [ (\kappa _1\!:\!\kappa _2) ] \! ]^{{ bi'}} \\ [ \! [ \lnot \varphi ] \! ]^{\tiny { KP}}= &  [ \! [ \lnot ] \! ]([ \! [ \varphi ] \! ]^{\tiny { KP}}), \ \ \small { \text{ where } [ \! [ \lnot ] \! ](0)\!=\!1, [ \! [ \lnot ] \! ](1)\!=\!0 \text{ and } [ \! [ \lnot ] \! ](*)\!=\!*} \\ [ \! [ \varphi \,{\textsf {op}}\,\psi ] \! ]^{\tiny { KP}}= &  [ \! [ {\textsf {op}} ] \! ]^{\tiny { KP}}([ \! [ \varphi ] \! ]^{\tiny { KP}},[ \! [ \psi ] \! ]^{\tiny { KP}}) \end{aligned}$$

With Definition A.3, all binary operators can be expressed using negation and conjunction. Specifically, for disjunction and implication we standardly get:

### Fact A.1

For any $\varphi ,\psi \in L_3$, for any KP-interpretation:

$$\begin{aligned} &  [ \! [ \varphi \vee \psi ] \! ]^{\tiny { KP}} = [ \! [ \lnot ((\lnot \varphi )\wedge \lnot \psi ) ] \! ]^{\tiny { KP}} \\ &  [ \! [ \varphi \rightarrow \psi ] \! ]^{\tiny { KP}} = [ \! [ \lnot (\varphi \wedge \lnot \psi ) ] \! ]^{\tiny { KP}} \end{aligned}$$

## Proofs of theorems

Theorem 1 below relies on the semantic notions of presuppositional conclusion and admittance condition, in relation to KP-interpretations. These notions of *KP-presupposition* and *KP-admittance* are formally defined below:

### Definition B.1

*(KP-presupposition)* A trivalent formula $\kappa \in L_3$ *KP-presupposes* bivalent formula $\alpha \in L_2$ if for any bivalent interpretation $[ \! [ \cdot ] \! ]^{{ bi}}$ and its corresponding KP-interpretation $[ \! [ \cdot ] \! ]^{\tiny { KP}}$:

if $$[ \! [ \kappa ] \! ]^{\tiny { KP}}\!\not =\!*$$ then $$[ \! [ \alpha ] \! ]^{{ bi}}\!=\!1$$.

In words: under any KP-interpretation, if $\kappa$ is well-defined then $\alpha$ is true.

### Definition B.2

*(KP-admittance)* A bivalent formula $\alpha \in L_2$ *KP-admits* a trivalent formula $\kappa \in L_3$ if for all KP-interpretations:

$$[ \! [ \alpha [\kappa ] ] \! ]^{\tiny { KP}}\!\not =*$$.

In words: in the context of $\alpha$, the formula $\kappa$ is well-defined under any KP-interpretation.

### Theorem 1

For any trivalent formula $\kappa \in L_3$, any strongest KP-presupposition $\alpha _1\in L_2$ of $\kappa$ is equivalent to any weakest $\alpha _2\in L_2$ that KP-admits $\kappa$. Thus, for any bivalent interpretation: $[ \! [ \alpha _1 ] \! ]^{{ bi}}\!=\!1 \ \ \ { iff} \ \ \ [ \! [ \alpha _2 ] \! ]^{{ bi}}\!=\!1$.

### Proof

By construction of $\kappa \in L_3$, there is a bivalent formula $\beta \in L_2$ s.t. for any KP-interpretation:

$$[ \! [ \beta ] \! ]^{{ bi}}\!=\!1 \ \ { iff} \ \ [ \! [ \kappa ] \! ]^{\tiny { KP}}\!\not =\!*$$ *(i)*

Since $\alpha _1$ is a KP-presupposition of $\kappa$, we have $[ \! [ \kappa ] \! ]^{\tiny { KP}}\!\not =\!*\Rightarrow \alpha _1$, hence by (i), $\beta \Rightarrow \alpha _1$. By (i), $\beta$ is also a KP-presupposition of $\kappa$. Thus, since $\alpha _1$ is a strongest KP-presupposition of $\kappa$ we have:

$$\alpha _1\equiv \beta $$ *(ii)*

Since $\alpha _2$ is a KP-admittance condition of $\kappa$, we have for any KP-interpretation:

$$[ \! [ \alpha _2[\kappa ] ] \! ]^{\tiny { KP}} = [ \! [ (\top \!:\!\alpha _2)\wedge \kappa ] \! ]^{\tiny { KP}} \not = *$$

We conclude by definition of KP-conjunction:

If $$[ \! [ \alpha _2 ] \! ]^{{ bi}}\!=\!1$$ then $$[ \! [ \kappa ] \! ]^{\tiny { KP}}\!\not =\!*$$.

Thus, by (i) we have: $\alpha _2\Rightarrow \beta$*(iii)*

By (i) and definition of KP-conjunction, we have for any KP-interpretation:

$$[ \! [ \beta [\kappa ] ] \! ]^{\tiny { KP}} = [ \! [ (\top \!:\!\beta )\wedge \kappa ] \! ]^{\tiny { KP}} \not = *$$

hence $\beta$ KP-admits $\kappa$*(iv)*

From (iii) and (iv), and since $\alpha _2$ is a weakest KP-admittance condition of $\kappa$ we have: $\alpha _2\equiv \beta$.

From (ii) we conclude: $\alpha _1\equiv \alpha _2$. $\square$

For proving Theorems 2 and 3, it is useful to note that, similarly to the KP tables (Fact A.1), the K-calculus standardly allows expressing disjunction and implication using negation and conjunction. Formally:

### Fact B.1

For any $\varphi ,\psi \in L_3$, for any $\alpha \in L_2$:

$$\begin{aligned} {\varvec{P}}^{\tiny {\text{ K }}}(\alpha [\varphi \vee \psi ])\equiv &  {\varvec{P}}^{\tiny {\text{ K }}}(\alpha [\lnot ((\lnot \varphi )\wedge \lnot \psi )]) \\ {\varvec{P}}^{\tiny {\text{ K }}}(\alpha [\varphi \rightarrow \psi ])\equiv &  {\varvec{P}}^{\tiny {\text{ K }}}(\alpha [\lnot (\varphi \wedge \lnot \psi )]) \end{aligned}$$

### Theorem 2

For any bivalent formula $\alpha \in L_2$ and trivalent formula $\kappa \in L_3$:

$\alpha$ K-admits $\kappa$*iff*$\alpha$ KP-admits $\kappa$.

### Proof

By soundness of the KP calculus (Fact 2.1), we need to show for any $\alpha \in L_2$ and $\kappa \in L_3$:

$${\varvec{P}}^{\tiny {\text{ K }}}(\alpha [\kappa ])\equiv \top $$ iff $${\varvec{P}}^{\tiny {\text{ KP }}}(\alpha [\kappa ])\equiv \top $$.

We will show that by induction on the structure of $\kappa$. Thus, for any $\alpha \in L_2$ and subformula $\kappa '$ of $\kappa$, we assume that $\alpha$ K-admits $\kappa '$ if $\alpha$ KP-admits $\kappa '$. By Facts A.1 and B.1, we only need to consider simple $L_3$ formulas and complex formulas $\kappa$ that are made of negation and conjunction:

$$\underline{\kappa =(\kappa _1\!:\!\kappa _2)}$$:

By definition of K-calculus:

$${\varvec{P}}^{\tiny {\text{ K }}}(\alpha [(\kappa _1\!:\!\kappa _2)])\equiv \top $$ iff $$\alpha \Rightarrow \kappa _1$$.

By definition of KP-calculus:

$${\varvec{P}}^{\tiny {\text{ KP }}}(\alpha [(\kappa _{1}\!:\!\kappa _{2})])\equiv \top $$ iff $${\varvec{P}}^{\tiny {\text{ KP }}}((\top \!:\!\alpha )\wedge (\kappa _{1}\!:\!\kappa _{2}))\equiv \top $$ iff $$\kappa _{1}\vee \lnot \alpha \equiv \top $$

iff $$\alpha \Rightarrow \kappa _1$$.

We conclude: ${\varvec{P}}^{\tiny {\text{ K }}}(\alpha [(\kappa _1\!:\!\kappa _2)])\equiv \top$ iff ${\varvec{P}}^{\tiny {\text{ KP }}}(\alpha [(\kappa _1\!:\!\kappa _2)])\equiv \top$.

$$\underline{\kappa =\lnot \varphi }$$:

By definition of K-calculus:

$${\varvec{P}}^{\tiny {\text{ K }}}(\alpha [\lnot \varphi ])={\varvec{P}}^{\tiny {\text{ K }}}(\alpha [\varphi ])$$.

By definition of KP-calculus:

$${\varvec{P}}^{\tiny {\text{ KP }}}(\alpha [\lnot \varphi ]) $$

$$={\varvec{P}}^{\tiny {\text{ KP }}}((\top \!:\!\alpha )\wedge \lnot \varphi )$$

$$ \equiv {\varvec{P}}^{\tiny {\text{ KP }}}(\lnot \varphi )\vee \lnot \alpha $$

$$ \equiv {\varvec{P}}^{\tiny {\text{ KP }}}(\varphi )\vee \lnot \alpha $$

$$ \equiv {\varvec{P}}^{\tiny {\text{ KP }}}((\top \!:\!\alpha )\wedge \varphi )$$

$$ ={\varvec{P}}^{\tiny {\text{ KP }}}(\alpha [\varphi ])$$

By induction ${\varvec{P}}^{\tiny {\text{ K }}}(\alpha [\varphi ])\equiv {\varvec{P}}^{\tiny {\text{ KP }}}(\alpha [\varphi ])$, hence we conclude: ${\varvec{P}}^{\tiny {\text{ K }}}(\alpha [\lnot \varphi ])\equiv {\varvec{P}}^{\tiny {\text{ KP }}}(\alpha [\lnot \varphi ])$.

$$\underline{\kappa =\varphi \wedge \psi }$$:

By definition of K-calculus:

$${\varvec{P}}^{\tiny {\text{ K }}}(\alpha [\varphi \wedge \psi ])= {\varvec{P}}^{\tiny {\text{ K }}}(\alpha [\varphi ]) \wedge {\varvec{P}}^{\tiny {\text{ K }}}((\alpha \wedge {\varvec{P}}^{\tiny {\text{ K }}}(\alpha [\varphi ])\wedge {\varvec{A}}(\varphi ))[\psi ])$$

Thus, we conclude:

$${\varvec{P}}^{\tiny {\text{ K }}}(\alpha [\varphi \wedge \psi ])\equiv \top $$

iff $${\varvec{P}}^{\tiny {\text{ K }}}(\alpha [\varphi ])\equiv \top $$ and $${\varvec{P}}^{\tiny {\text{ K }}}((\alpha \wedge {\varvec{P}}^{\tiny {\text{ K }}}(\alpha [\varphi ])\wedge {\varvec{A}}(\varphi ))[\psi ])\equiv \top $$

iff $${\varvec{P}}^{\tiny {\text{ K }}}(\alpha [\varphi ])\equiv \top $$ and $${\varvec{P}}^{\tiny {\text{ K }}}((\alpha \wedge {\varvec{A}}(\varphi ))[\psi ])\equiv \top $$

And by induction:

iff $${\varvec{P}}^{\tiny {\text{ KP }}}(\alpha [\varphi ])\equiv \top $$ and $${\varvec{P}}^{\tiny {\text{ KP }}}((\alpha \wedge {\varvec{A}}(\varphi ))[\psi ])\equiv \top $$*(i)*

We note:

$${\varvec{P}}^{\tiny {\text{ KP }}}(\alpha [\varphi \wedge \psi ])$$

$$= {\varvec{P}}^{\tiny {\text{ KP }}}((\top \!:\!\alpha )\wedge (\varphi \wedge \psi ))$$

$$\equiv {\varvec{P}}^{\tiny {\text{ KP }}}(\varphi \wedge \psi )\vee \lnot \alpha $$

$$\equiv ({\varvec{P}}^{\tiny {\text{ KP }}}(\varphi )\wedge ({\varvec{P}}^{\tiny {\text{ KP }}}(\psi )\vee \lnot {\varvec{A}}(\varphi )))\vee \lnot \alpha $$

$$\equiv ({\varvec{P}}^{\tiny {\text{ KP }}}(\varphi )\vee \lnot \alpha )\wedge (({\varvec{P}}^{\tiny {\text{ KP }}}(\psi )\vee \lnot {\varvec{A}}(\varphi ))\vee \lnot \alpha )$$

$$\equiv ({\varvec{P}}^{\tiny {\text{ KP }}}(\varphi )\vee \lnot \alpha ) \wedge ({\varvec{P}}^{\tiny {\text{ KP }}}(\psi )\vee \lnot (\alpha \wedge {\varvec{A}}(\varphi )))$$

$$\equiv {\varvec{P}}^{\tiny {\text{ KP }}}((\top \!:\!\alpha )\wedge \varphi ) \wedge {\varvec{P}}^{\tiny {\text{ KP }}}((\top \!:\!\alpha \wedge {\varvec{A}}(\varphi ))\wedge \psi )$$

$$= {\varvec{P}}^{\tiny {\text{ KP }}}(\alpha [\varphi ])\wedge {\varvec{P}}^{\tiny {\text{ KP }}}((\alpha \wedge {\varvec{A}}(\varphi ))[\psi ])$$

Thus:

$${\varvec{P}}^{\tiny {\text{ KP }}}(\alpha [\varphi \wedge \psi ])\equiv \top $$

iff $${\varvec{P}}^{\tiny {\text{ KP }}}(\alpha [\varphi ])\equiv \top $$ and $${\varvec{P}}^{\tiny {\text{ KP }}}((\alpha \wedge {\varvec{A}}(\varphi ))[\psi ])\equiv \top $$

And from (i) we conclude:

$${\varvec{P}}^{\tiny {\text{ K }}}(\alpha [\varphi \wedge \psi ])\equiv \top $$ iff $${\varvec{P}}^{\tiny {\text{ KP }}}(\alpha [\varphi \wedge \psi ])\equiv \top $$

$$\square $$

The following lemma is useful for proving Theorem 3:

### Lemma 1

For any trivalent $\kappa \!\in \!L_3$ and bivalent $\alpha ,\beta \!\in \!L_2$ s.t. $\alpha \!\Rightarrow \!\beta$ and $\beta$ K-admits $\kappa$, we have: $\alpha$ K-admits $\kappa$.

In words: K-admittance is closed under logical strengthening of the context.

### Proof

We will show that by induction on the structure of $\kappa$. Thus, for any subformula $\kappa '$ of $\kappa$, we assume that K-admittance of $\kappa '$ is closed under strengthening of the context. By Fact B.1, we only need to consider simple $L_3$ formulas and complex formulas $\kappa$ that are made of negation and conjunction:

$$\underline{\kappa =(\kappa _1\!:\!\kappa _2)}$$:

By assumption $\beta$ K-admits $(\kappa _1\!:\!\kappa _2)$, hence by definition of K-calculus: $\beta \Rightarrow \kappa _1$.

From $\alpha \!\Rightarrow \!\beta$ we conclude that $\alpha \!\Rightarrow \!\kappa _1$, hence by definition of K-calculus:

$$\alpha $$ K-admits $$(\kappa _1\!:\!\kappa _2)$$.

$$\underline{\kappa =\lnot \varphi }$$:

By assumption $\beta$ K-admits $\lnot \varphi$, hence by definition of K-calculus: $\beta$ K-admits $\varphi$.

From $\alpha \!\Rightarrow \!\beta$ we conclude by induction that $\alpha$ K-admits $\varphi$.

Thus, by definition of K-calculus $\alpha$ K-admits $\lnot \varphi$.

$$\underline{\kappa =\varphi \wedge \psi }$$:

By assumption $\beta$ K-admits $\varphi \wedge \psi$, hence by definition of K-calculus:

$$\begin{aligned} {\varvec{P}}^{\tiny {\text{ K }}}(\beta [\varphi \wedge \psi ]) \ \ = \ \ {\varvec{P}}^{\tiny {\text{ K }}}(\beta [\varphi ]) \wedge {\varvec{P}}^{\tiny {\text{ K }}}((\beta \wedge {\varvec{P}}^{\tiny {\text{ K }}}(\beta [\varphi ]) \wedge {\varvec{A}}(\varphi ))[\psi ]) \ \ \equiv \ \ \top \quad {(i)} \end{aligned}$$

From (i) we conclude that ${\varvec{P}}^{\tiny {\text{ K }}}(\beta [\varphi ])\equiv \top$, hence by induction, since $\alpha \!\Rightarrow \!\beta$:

$${\varvec{P}}^{\tiny {\text{ K }}}(\alpha [\varphi ])\equiv \top $$       *(ii)*

By substituting ${\varvec{P}}^{\tiny {\text{ K }}}(\beta [\varphi ])\equiv \top$ in (i), we get:

$$\begin{aligned} {\varvec{P}}^{\tiny {\text{ K }}}(\beta [\varphi \wedge \psi ]) \ \ \equiv \ \ \top \wedge {\varvec{P}}^{\tiny {\text{ K }}}((\beta \wedge \top \wedge {\varvec{A}}(\varphi ))[\psi ]) \ \ \equiv \ \ \top \end{aligned}$$

Or: $${\varvec{P}}^{\tiny {\text{ K }}}((\beta \wedge {\varvec{A}}(\varphi ))[\psi ]) \ \ \equiv \ \ \top $$

And by induction, since $\alpha \wedge {\varvec{A}}(\varphi )\Rightarrow \beta \wedge {\varvec{A}}(\varphi )$:

$${\varvec{P}}^{\tiny {\text{ K }}}((\alpha \wedge {\varvec{A}}(\varphi ))[\psi ]) \ \ \equiv \ \ \top $$       *(iii)*

By definition of K-calculus and (ii)–(iii), we conclude:

$${\varvec{P}}^{\tiny {\text{ K }}}(\alpha [\varphi \wedge \psi ])$$

$$ = {\varvec{P}}^{\tiny {\text{ K }}}(\alpha [\varphi ]) \wedge {\varvec{P}}^{\tiny {\text{ K }}}((\alpha \wedge {\varvec{P}}^{\tiny {\text{ K }}}(\alpha [\varphi ]) \wedge {\varvec{A}}(\varphi ))[\psi ])$$

$${\mathop {\equiv }\limits ^{{\tiny (ii )}}} \top \wedge {\varvec{P}}^{\tiny {\text{ K }}}((\alpha \wedge \top \wedge {\varvec{A}}(\varphi ))[\psi ]) $$

$${\mathop {\equiv }\limits ^{{\tiny (iii )}}} \top $$

Thus, $$\alpha $$ K-admits $$\varphi \wedge \psi $$.

$$\square $$

### Theorem 3

For any trivalent formula $\kappa \in L_3$:

$${\varvec{P}}^{\tiny {\text{ K }}}(\kappa )$$ K-admits $$\kappa $$.

### Proof

The proof will follow from substituting $\alpha =\top$ in a more general claim:

*For any trivalent formula* $\kappa \in L_3$ *and bivalent formula* $\alpha \in L_2$:

$${\varvec{P}}^{\tiny {\text{ K }}}((\alpha \wedge {\varvec{P}}^{\tiny {\text{ K }}}(\alpha [\kappa ]))[\kappa ]) \equiv \top $$, *i.e.,* $$\alpha \wedge {\varvec{P}}^{\tiny {\text{ K }}}(\alpha [\kappa ])$$ K-admits $$\kappa $$          *(i)*

To prove (i) for any $\alpha \in L_2$, we rely on Lemma 1 using an induction on the structure of $\kappa$. Thus, we assume that for any subformula $\kappa '$ of $\kappa$, for any $\alpha \in L_2$: $\alpha \wedge {\varvec{P}}^{\tiny {\text{ K }}}(\alpha [\kappa '])$ K-admits $\kappa '$. By Fact B.1, for this induction we only need to consider simple $L_3$ formulas and complex formulas $\kappa$ that are made of negation and conjunction:

$$\underline{\kappa =(\kappa _1\!:\!\kappa _2)}$$:

If $\alpha \Rightarrow \kappa _1$ then by definition of K-calculus: ${\varvec{P}}^{\tiny {\text{ K }}}(\alpha [(\kappa _1\!:\!\kappa _2)])=\top$. Thus:

$${\varvec{P}}^{\tiny {\text{ K }}}((\alpha \wedge {\varvec{P}}^{\tiny {\text{ K }}}(\alpha [(\kappa _1\!:\!\kappa _2)]))[(\kappa _1\!:\!\kappa _2)])\equiv {\varvec{P}}^{\tiny {\text{ K }}}((\alpha \wedge \top )[(\kappa _1\!:\!\kappa _2)])\equiv {\varvec{P}}^{\tiny {\text{ K }}}(\alpha [(\kappa _1\!:\!\kappa _2)])= \top $$.

If $\alpha \not \Rightarrow \kappa _1$ then by definition of K-calculus: ${\varvec{P}}^{\tiny {\text{ K }}}(\alpha [(\kappa _1\!:\!\kappa _2)])=\kappa _1$.

And since $$\alpha \wedge \kappa _1\Rightarrow \kappa _1$$:

$$ {\varvec{P}}^{\tiny {\text{ K }}}((\alpha \wedge {\varvec{P}}^{\tiny {\text{ K }}}(\alpha [(\kappa _1\!:\!\kappa _2)]))[(\kappa _1\!:\!\kappa _2)])\equiv {\varvec{P}}^{\tiny {\text{ K }}}((\alpha \wedge \kappa _1)[(\kappa _1\!:\!\kappa _2)])= \top $$.

$$\underline{\kappa =\lnot \varphi }$$:

By definition of K-calculus: ${\varvec{P}}^{\tiny {\text{ K }}}(\beta [\lnot \varphi ])={\varvec{P}}^{\tiny {\text{ K }}}(\beta [\varphi ])$ for any $\beta \in L_2$.

Thus:

$${\varvec{P}}^{\tiny {\text{ K }}}((\alpha \wedge {\varvec{P}}^{\tiny {\text{ K }}}(\alpha [\lnot \varphi ]))[\lnot \varphi ])$$

$$= {\varvec{P}}^{\tiny {\text{ K }}}((\alpha \wedge {\varvec{P}}^{\tiny {\text{ K }}}(\alpha [\varphi ]))[\lnot \varphi ])$$

$$= {\varvec{P}}^{\tiny {\text{ K }}}((\alpha \wedge {\varvec{P}}^{\tiny {\text{ K }}}(\alpha [\varphi ]))[\varphi ])$$

By the induction hypothesis:

$$\equiv \top $$

$$\underline{\kappa =\varphi \wedge \psi }$$:

By definition of K-calculus:

$${\varvec{P}}^{\tiny {\text{ K }}}(\alpha [\kappa ])={\varvec{P}}^{\tiny {\text{ K }}}(\alpha [\varphi \wedge \psi ])$$

      $$={\varvec{P}}^{\tiny {\text{ K }}}(\alpha [\varphi ])\wedge {\varvec{P}}^{\tiny {\text{ K }}}((\alpha \wedge {\varvec{P}}^{\tiny {\text{ K }}}(\alpha [\varphi ])\wedge {\varvec{A}}(\varphi ))[\psi ])$$

We denote:

      $$\theta = \alpha \wedge {\varvec{P}}^{\tiny {\text{ K }}}(\alpha [\kappa ]) = \alpha \wedge {\varvec{P}}^{\tiny {\text{ K }}}(\alpha [\varphi ])\wedge {\varvec{P}}^{\tiny {\text{ K }}}((\alpha \wedge {\varvec{P}}^{\tiny {\text{ K }}}(\alpha [\varphi ])\wedge {\varvec{A}}(\varphi ))[\psi ])$$*(ii)*

Thus: $${\varvec{P}}^{\tiny {\text{ K }}}((\alpha \wedge {\varvec{P}}^{\tiny {\text{ K }}}(\alpha [\kappa ]))[\kappa ]) \ = \ {\varvec{P}}^{\tiny {\text{ K }}}(\theta [\kappa ]) \ = \ {\varvec{P}}^{\tiny {\text{ K }}}(\theta [\varphi \wedge \psi ])$$

Thus, by definition of K-calculus, we have:

$${\varvec{P}}^{\tiny {\text{ K }}}((\alpha \wedge {\varvec{P}}^{\tiny {\text{ K }}}(\alpha [\kappa ]))[\kappa ]) \ = \ {\varvec{P}}^{\tiny {\text{ K }}}(\theta [\varphi ])\wedge {\varvec{P}}^{\tiny {\text{ K }}}((\theta \wedge {\varvec{P}}^{\tiny {\text{ K }}}(\theta [\varphi ])\wedge {\varvec{A}}(\varphi ))[\psi ])$$ *(iii)*

Now, from the induction hypothesis we have:

$${\varvec{P}}^{\tiny {\text{ K }}}((\alpha \wedge {\varvec{P}}^{\tiny {\text{ K }}}(\alpha [\varphi ]))[\varphi ])\!\equiv \!\top $$

Thus, by Lemma 1 we conclude for any bivalent formula $\tau \in L_2$:

$${\varvec{P}}^{\tiny {\text{ K }}}((\alpha \wedge {\varvec{P}}^{\tiny {\text{ K }}}(\alpha [\varphi ])\wedge \tau )[\varphi ]) \ \equiv \ \top $$

By substituting $\tau ={\varvec{P}}^{\tiny {\text{ K }}}((\alpha \wedge {\varvec{P}}^{\tiny {\text{ K }}}(\alpha [\varphi ])\wedge {\varvec{A}}(\varphi ))[\psi ])$, we have by our notation (ii):

$$\alpha \wedge {\varvec{P}}^{\tiny {\text{ K }}}(\alpha [\varphi ])\wedge \tau =\theta $$

Thus:

$${\varvec{P}}^{\tiny {\text{ K }}}(\theta [\varphi ])\equiv \top $$ *(iv)*

By substituting (iv) in (iii), we conclude:

$$\begin{aligned} {\varvec{P}}^{\tiny {\text{ K }}}((\alpha \wedge {\varvec{P}}^{\tiny {\text{ K }}}(\alpha [\kappa ]))[\kappa ]) \ \equiv \ \top \wedge {\varvec{P}}^{\tiny {\text{ K }}}((\theta \wedge \top \wedge {\varvec{A}}(\varphi ))[\psi ]) \ \equiv \ {\varvec{P}}^{\tiny {\text{ K }}}((\theta \wedge {\varvec{A}}(\varphi ))[\psi ]) \end{aligned}$$

By the notation in (ii):

$$={\varvec{P}}^{\tiny {\text{ K }}}((\alpha {\,\wedge \,{\varvec{P}}^{\tiny {\text{ K }}}(\alpha [\varphi ])}\wedge {\varvec{P}}^{\tiny {\text{ K }}}((\alpha \wedge {\varvec{P}}^{\tiny {\text{ K }}}(\alpha [\varphi ])\wedge {\varvec{A}}(\varphi ))[\psi ])\wedge {\varvec{A}}(\varphi ))[\psi ])$$

By denoting $\alpha _0=\alpha {\,\wedge \,{\varvec{P}}^{\tiny {\text{ K }}}(\alpha [\varphi ])}\wedge {\varvec{A}}(\varphi )$, we get:

$$={\varvec{P}}^{\tiny {\text{ K }}}((\alpha _0\wedge {\varvec{P}}^{\tiny {\text{ K }}}(\alpha _0[\psi ]))[\psi ])$$

$$\equiv \top $$ by induction.

We have proven Fact (i) above, from which we conclude that for any formula $\kappa \in L_3$:

$$ {\varvec{P}}^{\tiny {\text{ K }}}((\top \wedge {\varvec{P}}^{\tiny {\text{ K }}}(\top [\kappa ]))[\kappa ]) \ \equiv \ \top $$

Thus, $${\varvec{P}}^{\tiny {\text{ K }}}(({\varvec{P}}^{\tiny {\text{ K }}}(\kappa ))[\kappa ])\equiv \top $$, or:

$${\varvec{P}}^{\tiny {\text{ K }}}(\kappa )$$ K-admits $$\kappa $$. $$\square $$

**Fact **  2.2. For all trivalent formulas $\varphi ,\psi$ in $L_3$:

$$\begin{aligned} {\varvec{P}}^{\tiny {\text{ KP }}}(\varphi \,{\textsf {op}}\,\psi ) \equiv {\varvec{P}}^{\tiny {\text{ KP }}}(\varphi )\,\wedge \,{\varvec{P}}^{\tiny {\text{ KP }}}(({\varvec{P}}^{\tiny {\text{ KP }}}(\varphi )\!\wedge \!\lnot {\textsc {ldv}}_{{\small {\textsf {op}}}}({\varvec{A}}(\varphi )))[\psi ]) \end{aligned}$$

**Proof**

$$\square $$

## Footnotes

1 For further discussion of Karttunen (1974) and other dynamic approaches, see Francez (2019).

2 The term “previously processed” refers to configurational parsing, meaning that the order of interpretation does not necessarily correspond with linear order (Mandelkern & Romoli, 2017; Chung, 2018). This point does not affect the examples presented in this paper.

3 For the purposes of the discussion in this paper, we treat conditionals as material implications, setting aside potential complications that might affect the analysis of presuppositions (Pérez Carballo, 2008).

4 Since *C* is bivalent, the KP conjunction of *C* and (11) is well-defined if and only if either *C* is false or (11) is well-defined. Using KP implication, the latter condition is equivalent to the requirement that (12) is true. Thus, the conjunction of *C* and (11) is well-defined in all situations if and only if *C* entails (12).

5 Peters concluded that this congruence undermines Karttunen’s argument against truth-functional accounts. However, Peters’s semantics did not correctly reflect Karttunen’s entailment-based analysis, and ignored his empirical argument against classical trivalent semantics: the proviso problem (Karttunen 1973, p. 188).

6 See Heim (1982, 1992), Beaver (2001), Rothschild (2011), Nouwen et al. (2016), and references therein. Another notable early dynamic proposal is Heim (1983), which, however, is not committed to interpreting Karttunen’s system using possible worlds.

7 For recent proposals and experimental results about (a)symmetric projection, see Kalomoiros (2023) and the references therein.

8 Due to the following properties of Kleene systems, this property is shared by all trivalent truth tables in the “Kleene hierarchy” (Fitting, 1994):

(i) If $[ \! [ \varphi ] \! ]\not =*$ and $[ \! [ \psi ] \! ]\not =*$, then $[ \! [ \varphi \,{\textsf {op}}\,\psi ] \! ]\not =*$
  (if the presuppositions of both operands are satisfied, then the presuppositions of the binary construction are satisfied as well).
(ii) If $[ \! [ \varphi ] \! ]=*$ and $[ \! [ \varphi \,{\textsf {op}}\,\psi ] \! ]\not =*$, then $[ \! [ \varphi \,{\textsf {op}}\,\psi ] \! ]=[ \! [ \bot \,{\textsf {op}}\,\psi ] \! ]=[ \! [ \top \,{\textsf {op}}\,\psi ] \! ]$;
  If $[ \! [ \psi ] \! ]=*$ and $[ \! [ \varphi \,{\textsf {op}}\,\psi ] \! ]\not =*$, then $[ \! [ \varphi \,{\textsf {op}}\,\psi ] \! ]=[ \! [ \varphi \,{\textsf {op}}\,\bot ] \! ]=[ \! [ \varphi \,{\textsf {op}}\,\top ] \! ]$
  (if one operand shows a presupposition failure but the presuppositions of the binary construction are satisfied, then the value of the other operand determines the result of the operation, in the sense defined below).

9 This does not add to the expressivity of the K-calculus compared to the KP calculus. We could equivalently introduce the K-calculus using the item form $(\top \!:\!\alpha )\wedge \kappa$ in $L_3$ instead of its “syntactic sugaring” $\alpha [\kappa ]$. Incidentally, the Heim-Stalnaker’s way of updating contexts inductively uses forms $(\ldots ((\alpha [\kappa _{1}])[\kappa _{2}])\ldots )[\kappa _{n}]$, where $\kappa _1, \kappa _2,\ldots$ are trivalent. This is a sugaring for $(\top \!:\!\alpha )\wedge \kappa _{1}\wedge \kappa _{2}\wedge \ldots \wedge \kappa _{n}$.

10 Most adaptations of Karttunen (1974) describe contexts and lexical presuppositions as sets of possible worlds (Stalnaker, 1973), thus are less restrictive than entailment. Outside DRT (van der Sandt, 1988), entailments between formulas were rarely explored until Mandelkern (2016a).

11 I am thankful to an *L&P* reviewer for remarks in relation to this conjecture.

12 In (40)’s consequent, the factive’s complement, a known presupposition trigger, has a non-trivial presupposition (‘Sue used to smoke’). In such cases, Beaver & Krahmer (2001, p. 150) propose a recursive definition where presuppositions are trivalent, i.e., can have non-trivial presuppositions (cf. Blamey’s 1986 transplication). Here, we ignore this complexity and treat sentences like *Dan knows Sue continues to smoke* as presupposing the bivalent conjunction of the complement’s presupposition (‘Sue used to smoke’) and its assertive component (‘Sue smokes’).

13 Famously, the assertive meaning of the verb *know* is more complicated than ‘to believe something true’. This hardly affects the current treatment, as $B_{{\tiny {S }}}$ can be interpreted using other analyses of *know*.

14 (43) mirrors the point made earlier with (30a). Karttunen (1973, p. 188) first noted this issue for trivalent theories; Geurts (1996) later called it the “proviso problem” for possible-world theories of satisfaction.

15 Since the early proposals by Karttunen and Peters (1979) and Soames (1982), different accounts have been proposed as to the origins of strengthening: see van Rooij (2007), Singh (2007), Schlenker (2011), Lassiter (2012), Fox (2013, 2022), and Mayr and Romoli (2016), among others. For critique, see Geurts (1996), Mandelkern (2016a, 2016b, 2018) and Mandelkern and Rothschild (2018).

16 Similar examples have been discussed in the literature since Karttunen (1973, p. 184, 1974, p. 192). Sentence (48) is a minor variation on similar examples from Karttunen (1974) and Schlenker (2007).

17 This pragmatic direction was initiated by Karttunen (1973, 1974), and followed in various forms in Gazdar (1979), van der Sandt (1988), Geurts (1996, 1999), and Mandelkern (2016a), among others.

18 On logical inferences from generics like (51), see Veltman (1996), among others.

19 Such a defeat does not necessarily lead to logically weaker statement than *p*. For example, in relation to (53), a plausible candidate for K-accommodation is (56), which is logically independent of the K-presupposition (55).

20 See Mandelkern (2016a,2016b) for additional examples that illustrate the same point.

21 I am thankful to an *L&P* reviewer for pointing out such examples.
